# From extracellular entry to intracellular release: A water‐assisted transport cycle for creatine in SLC6A8

**DOI:** 10.1002/pro.70671

**Published:** 2026-06-18

**Authors:** Pitambar Poudel, Shailesh Kumar Panday, Emil Alexov

**Affiliations:** ^1^ Department of Physics & Astronomy, College of Science Clemson University Clemson South Carolina USA; ^2^ Medical Biophysics Program Clemson University Clemson South Carolina USA; ^3^ Center for Human Genetics Clemson University Greenwood South Carolina USA

**Keywords:** creatine transporter, membrane transport, molecular dynamics, SLC6A8, solute carrier, water‐mediated mechanism

## Abstract

The creatine transporter (CRT/SLC6A8) plays a key role in cellular energy homeostasis, yet the molecular mechanism underlying creatine transport remains poorly understood. Here, we reconstruct the complete transport cycle of human CRT using a hybrid simulation strategy that combines constant‐force steered molecular dynamics (cf‐sMD) with targeted molecular dynamics (tMD). This approach captures continuous progression through the outward‐open, outward‐occluded, inward‐occluded, and inward‐open states and reveals a water‐assisted, sequential intracellular release of Na2, creatine, and Na1. Hydration analysis shows that progressive water penetration into the binding pocket weakens protein‐substrate and protein‐ion interactions and destabilizes the bound state before release. Residue‐level contact analysis identifies residues that interact with creatine along the transport pathway, while dynamic network analysis reveals a TM1–TM6 communication backbone that mediates long‐range coupling during transport. Together, these results provide a molecular framework for creatine transport and establish an approach for investigating transport mechanisms across the broader solute carrier family.

## 1 INTRODUCTION

Solute carriers (SLCs) form the largest family of secondary active transporters in the human genome, with over 400 members organized into 65 families. These transporters facilitate the transmembrane uptake and exchange of various solutes, including inorganic ions, amino acids, sugars, lipids, neurotransmitters, and drugs (He et al., 2009; Hediger et al., 2004; Liu, 2019). Through these functions, SLC proteins help maintain nutrient balance, regulate signaling pathways, and support energy homeostasis. Among them, the creatine transporter (CRT), encoded by the *SLC6A8* gene, is particularly important due to its physiological role and involvement in human disorders (Mulik & Mercimek‐Andrews, 2023; Nasrallah et al., 2010). SLC6A8 belongs to the SLC6 family of SLCs, also known as neurotransmitter: sodium symporters (NSS) or the Na^+^/Cl^−^‐dependent transporter family (Beuming et al., 2006; Mulik & Mercimek‐Andrews, 2023), and is responsible for importing creatine into high‐energy‐demand tissues such as the brain, heart, and skeletal muscle (Béard & Braissant, 2010; Schloss et al., 1994). Creatine is a guanidinium‐based compound that acts as a rapid, reversible cellular energy buffer by storing high‐energy phosphate bonds (Wyss & Kaddurah‐Daouk, 2000). In its phosphorylated form (phosphocreatine), it donates a phosphate group to ADP, regenerating ATP and helping stabilize cellular ATP levels during periods of high energy demand via the creatine‐phosphocreatine shuttle (Joncquel‐Chevalier Curt et al., 2015; Kreider & Stout, 2021). The importance of CRT is also highlighted by the consequences of its deficiency. Insufficient creatine supply disrupts muscle and nervous tissue development and function, leading to cerebral creatine deficiency syndrome (CCDS) (Schulze, 2003; van de Kamp et al., 2013). In humans, CCDS can result from impaired biosynthesis or defective transport, including guanidinoacetate methyltransferase (GAMT) deficiency, arginine‐glycine amidinotransferase (AGAT) deficiency, and creatine transporter deficiency (CTD) (Nasrallah et al., 2010; Salomons et al., 2001). While the first two reduce creatine production and can often be treated with dietary supplementation, mutations in *SLC6A8* impair creatine uptake and lead to CTD, the most common and severe form of CCDS (Salomons et al., 2001). CTD is an X‐linked disorder associated with intellectual disability and other neurological symptoms, accounting for 1%–2% of unexplained X‐linked intellectual disabilities (Ferrada et al., 2024; Lion‐Francois et al., 2006). These observations clearly highlight the importance of CRT function and the need for a mechanistic understanding of creatine transport. Indeed, understanding the molecular mechanisms behind creatine transport will help interpret pathogenic mutations and may aid in developing targeted therapies.

Despite its clinical importance, the molecular mechanisms by which CRT transports creatine remain incompletely understood, despite extensive study of members of the SLC family. The NSS family comprises secondary active transporters that couple substrate movement with ion gradients (Gu et al., 1994; Kanner & Zomot, 2008; Torres et al., 2003). The transported substrates include dopamine, serotonin, norepinephrine, the inhibitory neurotransmitter γ‐aminobutyric acid (GABA), amino acids such as glycine, proline, taurine, and leucine, and osmolytes such as betaine and creatine (Bröer & Gether, 2012). Structurally, these transporters share the LeuT fold, first identified in the bacterial leucine transporter LeuT, which was the first NSS transporter to be crystallographically resolved and characterized in multiple conformations (Krishnamurthy & Gouaux, 2012; Piscitelli et al., 2010; Singh et al., 2008; Yamashita et al., 2005). By adopting the LeuT fold, these transporters operate through the alternating‐access mechanism (Figure S1, Data S1, Supporting Information) (Forrest et al., 2011; Jardetzky, 1966). In this model, NSS transporters cycle through outward‐open, outward‐occluded, inward‐occluded, and inward‐open conformations (Figure S1, Data S1). In CRT, the outward‐open conformation is stabilized by two Na^+^ ions occupying the primary binding sites (Na1 and Na2) together with a Cl^−^ ion at its conserved site, while the primary substrate‐binding site (S1) remains unoccupied. In this state, the extracellular (EC) gate is open, and the intracellular (IC) gate is closed. The binding of creatine at the S1 site stabilizes the outward‐occluded conformation, in which the EC gate is nearly closed while the IC gate remains closed. Transition to the inward‐occluded conformation follows, characterized by a fully closed EC gate and a partially open IC gate. Finally, in the inward‐open conformation, creatine and the two Na^+^ ions are released into the cytoplasm, while the Cl^−^ ion remains bound at its conserved site (Chen et al., 2025). More than 150 structures adopting the LeuT fold have now been determined, providing insight into the structural basis of transport in this family (Forrest et al., 2008; Jardetzky, 1966). Structurally, the LeuT fold comprises 12 transmembrane helices (TM1–TM12) arranged as two pseudo‐symmetric inverted repeats (TM1–TM5 and TM6–TM10). TM1 and TM6 are each broken near the S1 region, forming the TM1a/TM1b and TM6a/TM6b segments. The structure is further divided into a scaffold domain (TM3, TM4, TM8, TM9) and a bundle domain (TM1, TM2, TM6, TM7), with substrate transport driven by the movement of the bundle relative to the scaffold domain (Kristensen et al., 2011), often described as the rocking‐bundle mechanism (Forrest & Rudnick, 2009). This motion enables alternating access to the conserved central binding site (S1) (Yamashita et al., 2005).

In several NSS members, including the leucine transporter (LeuT) (Celik et al., 2008; Cheng & Bahar, 2013; Shi et al., 2008; Thomas et al., 2012), dopamine transporter (DAT) (Cheng & Bahar, 2015; Shan et al., 2011), serotonin transporter (SERT) (Gradisch et al., 2022; Hellsberg et al., 2019; Koldsø et al., 2011), glutamate transporter (Glt) (Gu et al., 2009), and GABA transporters (GAT) (Nayak et al., 2023; Zhu et al., 2023), the alternating‐access mechanism has been extensively studied. Since CRT shares the same LeuT structural fold, it is expected to operate through a similar mechanism (Drew & Boudker, 2016). Supporting this, several computational studies have explored different aspects of CRT transport. A recent simulation study docked creatine into the modeled outward‐open conformation of CRT and observed progression toward an occluded conformation (Clarke et al., 2024). More recently, crystal structures of CRT in inward‐occluded and inward‐open conformations were solved, providing structural evidence for the alternating access (Chen et al., 2025), and a short MD simulation in the same study focused on creatine's stability in the binding pocket. In another study, creatine was docked directly into the binding pocket (S1), and binding poses across different conformations were analyzed without simulating the full transport cycle (Colas et al., 2020). Likewise, in another study, creatine was docked into the S1 site of a modeled outward‐open CRT, followed by microsecond‐scale unbiased MD simulations that captured a transition toward an occluded conformation but did not reproduce the full transport cycle (Clarke et al., 2024).

Despite advances in CRT and other NSS members, a detailed molecular understanding of creatine transport through CRT remains lacking. This is likely because unbiased molecular dynamics simulations are typically too slow to capture the large conformational changes necessary for substrate transport. While enhanced sampling methods can accelerate rare events, they do not replicate the driving force provided by the electrochemical gradient, which supplies the energy for substrate transport in NSS or SL6 family members. Since constant‐force‐steered MD (cf‐sMD) provides a good approximation of the electrochemical gradient's role, we used cf‐sMD to model creatine transport in CRT. However, cf‐sMD alone could not induce transitions between key conformational states, so we combined it with targeted MD (tMD) to create a hybrid strategy that maintains substrate‐guided motion while promoting realistic conformational changes. In this study, we employed this protocol to reveal, for the first time, the complete creatine transport pathway of wild‐type CRT. The study also involved addressing several key questions, including choosing the appropriate magnitude of the steering force to mimic the electrochemical gradient without causing unrealistic deformation of creatine or the transporter. Additionally, we focused on modeling all four relevant conformational states, which had not been previously accomplished. The selection of simulation duration and steering force magnitude was based on the expected sequence of creatine and sodium ion release. Indeed, the protocol successfully simulated the correct sequential release of Na2, followed by creatine, and then Na1 (Na2 → creatine → Na1). By developing this protocol and selecting appropriate parameters, we provide a broadly applicable framework for studying substrate transport in other members of the NSS family.

## 2 RESULTS

### 2.1 Selection of the magnitude of the steering force applied to creatine and ions over the conformational states

**FIGURE 1:**
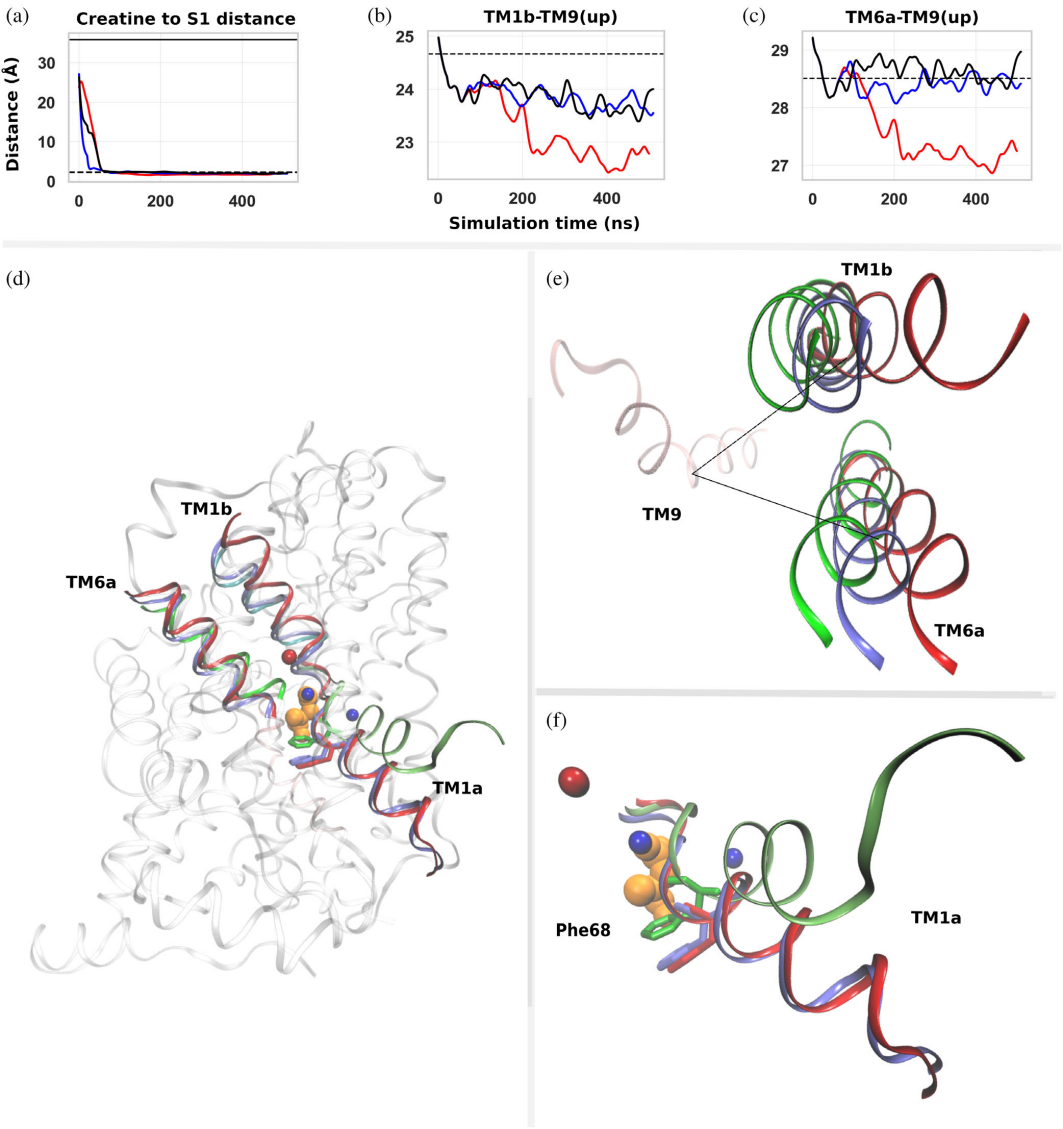
Creatine entry into the S1 site and conformational changes of TM1b, TM6a, and TM1a during cf‐sMD simulations. Distances and structural comparisons describing creatine transport toward the S1 binding site and the relative conformations of TM helices. (a) Distance between the center of mass (COM) of creatine and residues defining the S1 binding site during three independent simulations (black, blue, and red). The solid horizontal line indicates the corresponding distance in the outward‐open homology model used as the starting structure, and the dashed line indicates the distance observed in the inward‐occluded crystal structure (PDB 9KRH) shown for reference. (b) COM distance between TM1b and the upper segment of TM9 (TM9(up)) during the simulations. (c) COM distance between TM6a and TM9(up) during the simulations. For panels (a–c), the x‐axis represents simulation time (ns), and the y‐axis represents distance (Å). Traces correspond to locally averaged distances calculated using a 1 ns sliding window. Simulations were performed for 500 ns. (d) Structural overview of the creatine transporter (CRT), highlighting TM1b, TM6a, and TM1a, shown as ribbon representations. Creatine is shown as orange spheres, sodium ions as blue spheres, and chloride ions as red spheres. (e) Superposition of TM1b and TM6a from three structural states: the equilibrated outward‐open homology model used as the starting structure (red), the inward‐occluded crystal structure (green; PDB 9KRH), and the conformation obtained at the end of the cf‐sMD simulation (blue). (f) Superposition of TM1a across the same three conformational states shown in (e), with Phe68 shown in stick representation.

As mentioned above, the steering force in the protocol mimics the electrochemical gradient. Because this gradient is unknown, the first step was to determine the optimal magnitude of the steering force. We began with cf‐sMD simulations applying forces from 5 to 100 pN (500 ns, three independent replicas). Creatine was manually placed in the EC vestibule of the outward‐open conformation of CRT about 36 Å from the primary substrate‐binding site (S1), along the membrane normal (Figure S2, Data S1), and the steering force was initially applied only to creatine, while sodium and chloride ions remained at their binding sites. At 5 pN, creatine did not reach the S1 pocket within 500 ns in any replica (Figure S3A, Data S1), indicating that this force is insufficient to drive substrate entry over the simulated timescale. At 10 pN, creatine reached S1 within ~50 ns in all replicas but did not consistently induce EC gate closure or outward occlusion (Figure S3B, D, F, Data S1). Conversely, at 20 pN, creatine rapidly entered S1 in all replicas and remained stably bound for the remainder of the simulations (Figure 1a). Under this condition, both TM1b‐TM9(up) and TM6a‐TM9(up) distances decreased relative to the outward‐open starting structure, indicating progression toward outward occlusion (Figure 1b, c, Figure S3E, G, Data S1). The most pronounced closure occurred in replica 3 at 20 pN, where the TM1b‐TM9(up) distance decreased from ~24.6 to ~22.5 Å and remained reduced after ~200 ns, while the TM6a‐TM9(up) distance showed a smaller decrease from ~28.5 to ~27 Å. These results suggest TM1b plays a more prominent role than TM6a in early extracellular gate closure in CRT, consistent with previous observations in hSERT (Gradisch et al., 2022). Structural comparison supports this interpretation. Superposition of the conformation sampled at 400 ns in replica 3 with the equilibrated outward‐open conformation and the inward‐occluded crystal structure (PDB 9KRH) (Chen et al., 2025) shows that TM1b and TM6a shift away from the outward‐open conformation toward the inward‐occluded state, although the overlap is incomplete (Figure 1d, e). TM1b undergoes the larger displacement, supporting its role as the primary extracellular gating helix during early occlusion. Importantly, the EC gate closure observed in replica 3 at 20 pN is comparable to that reported for the outward‐occluded conformation of hSERT, where similar reductions in TM1b–TM9 and TM6a–TM9 distances define outward occlusion (Gradisch et al., 2022). Another study interpreted a TM1b–TM9 distance <26.0 Å in CRT as indicative of outward occlusion (Clarke et al., 2024); in our simulations, the reduction was even more pronounced and sustained, suggesting that the conformation sampled at 20 pN represents an outward‐occluded‐like state of CRT.

Despite successful creatine binding and outward occlusion at 20 pN, no transition toward inward‐occluded or inward‐open conformations occurred within the 500 ns simulation window. In particular, TM1a did not adopt the expected inward bend, and Phe68 did not undergo the rotational rearrangement associated with intracellular gating (Figure 1f). Increasing the force beyond 20 pN transported the substrate toward the cytosolic side but without the coordinated conformational transitions expected for physiological transport. Lower forces, therefore, failed to drive occlusion, whereas higher forces distorted the process. Taken together, these results identify 20 pN as the optimal force to drive creatine entry into S1 and promote outward occlusion while preserving realistic transporter dynamics. However, cf‐sMD alone remained insufficient to overcome the conformational barrier separating outward‐occluded and inward‐occluded/open states.

**FIGURE 2:**
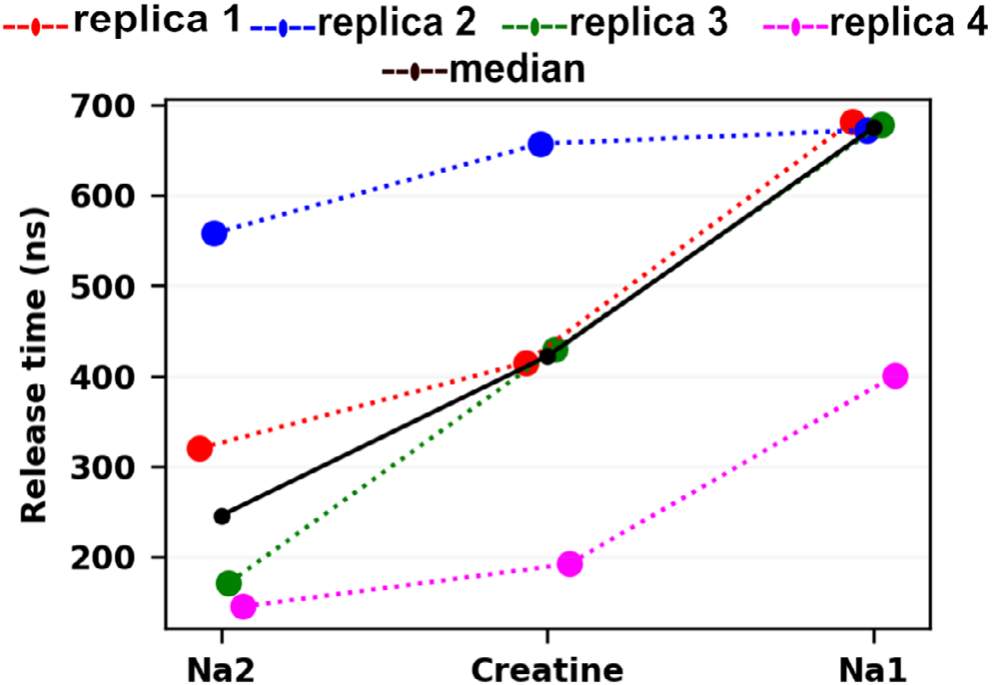
Release times for Na2, creatine, and Na1 during the 5 pN release stage of the hybrid cf‐sMD and tMD protocol. Release times (ns) measured from the start of targeted molecular dynamics (tMD), with *t* = 0 corresponding to tMD initiation from the 150 ns outward‐occluded starting structure. Colored dotted lines connect sequential release events within each replica (replicas 1–4), and the black line shows the median release time across replicas 1–4 at each step. The corresponding numerical values are provided in Table S1 in Data S1.

Building on these observations, we conducted exploratory simulations and chose the final protocol reported here, in which cf‐sMD and tMD were applied simultaneously. Since 20 pN transported creatine to S1 within approximately 50 ns and outward occlusion occurred in replica 3, the conformation of replica 3 at 150 ns was used as the starting point for subsequent hybrid simulations. Details of the exploratory simulations are provided in Data S1 (“All simulation trials”). From this 150 ns intermediate, both cf‐sMD and tMD were applied simultaneously in two stages: a 50 ns simulation targeting the inward‐occluded crystal structure (PDB 9KRH) (Chen et al., 2025), followed by a 50 ns simulation targeting the inward‐open crystal structure (PDB 9KR7) (Chen et al., 2025). Because this hybrid approach guided the system past the remaining conformational barriers, lower forces were also tested during the release stage to evaluate substrate and ion release. Simultaneous steering was applied to creatine and both Na^+^ ions with forces of 5, 10, and 20 pN. Since this protocol resulted in discrete intracellular release events for Na2, creatine, and Na1, the number of replicates was increased from three to five per force condition to assess the reproducibility of release order and timing (Figure 2; Table S1, Data S1). Across the tested force conditions, 5 pN most consistently produced the expected sequential release of the species (Na2, creatine, and Na1). In contrast, 10 and 20 pN more frequently produced either non‐sequential behavior (e.g., early creatine release before Na2) or incomplete release within the simulation window, indicating that higher forces can disrupt the expected pathway rather than simply accelerate it. These observations support the selection of 5 pN for the subsequent production simulations. Accordingly, all subsequent analyses of intracellular release focus on the 5 pN trajectories.

### 2.2 Release times of creatine and ions from the binding pocket to the intracellular side

Next, we quantify the intracellular release process at 5 pN by reporting the release times of Na2, creatine, and Na1, with Na1 release marking completion of the intracellular release stage (Figure 2; Table S1, Data S1). Release times were measured from the start of the tMD protocol (*t* = 0 at tMD initiation) using the 150 ns outward‐occluded starting structure. Across the five trajectories simulated at 5 pN, intracellular release followed the expected sequential order, although the complete release time varied between trajectories. In replicas 1–4, Na2 was released between 145 and 558 ns, creatine between 192 and 657 ns, and Na1 between 401 and 682 ns. The corresponding median release times were 246 ns for Na2, 422 ns for creatine, and 675 ns for Na1. In replica 5, only Na2 was released (180 ns), while creatine and Na1 remained bound within the simulated time window. This suggests that release order may reflect different kinetic barriers rather than a deterministic coupling mechanism. Nevertheless, while absolute release times varied across trajectories, the release order remained invariant in replicas 1–4. These results indicate that the hybrid protocol reliably places the transporter in an inward‐open‐like geometry and promotes the expected release pathway. We also analyzed the intervals separating successive release steps. In most trajectories, the Na2 → creatine interval ranged from ~47 to 99 ns, indicating that creatine release often followed Na2 release within a relatively short time window. In contrast, the creatine → Na1 interval was typically longer (~200–270 ns), suggesting that Na1 release frequently represents the final step of the intracellular release process. Together, these observations indicate that the variability between trajectories arises primarily from differences in the lifetimes of intermediate states rather than changes in release order. A detailed analysis of release timing and inter‐event intervals is provided in Data S1 (Trial 4: Continuous tMD with simultaneous cf‐sMD [final protocol]).

### 2.3 Hydration analysis reveals a water‐assisted release model of creatine and ions

**FIGURE 3:**
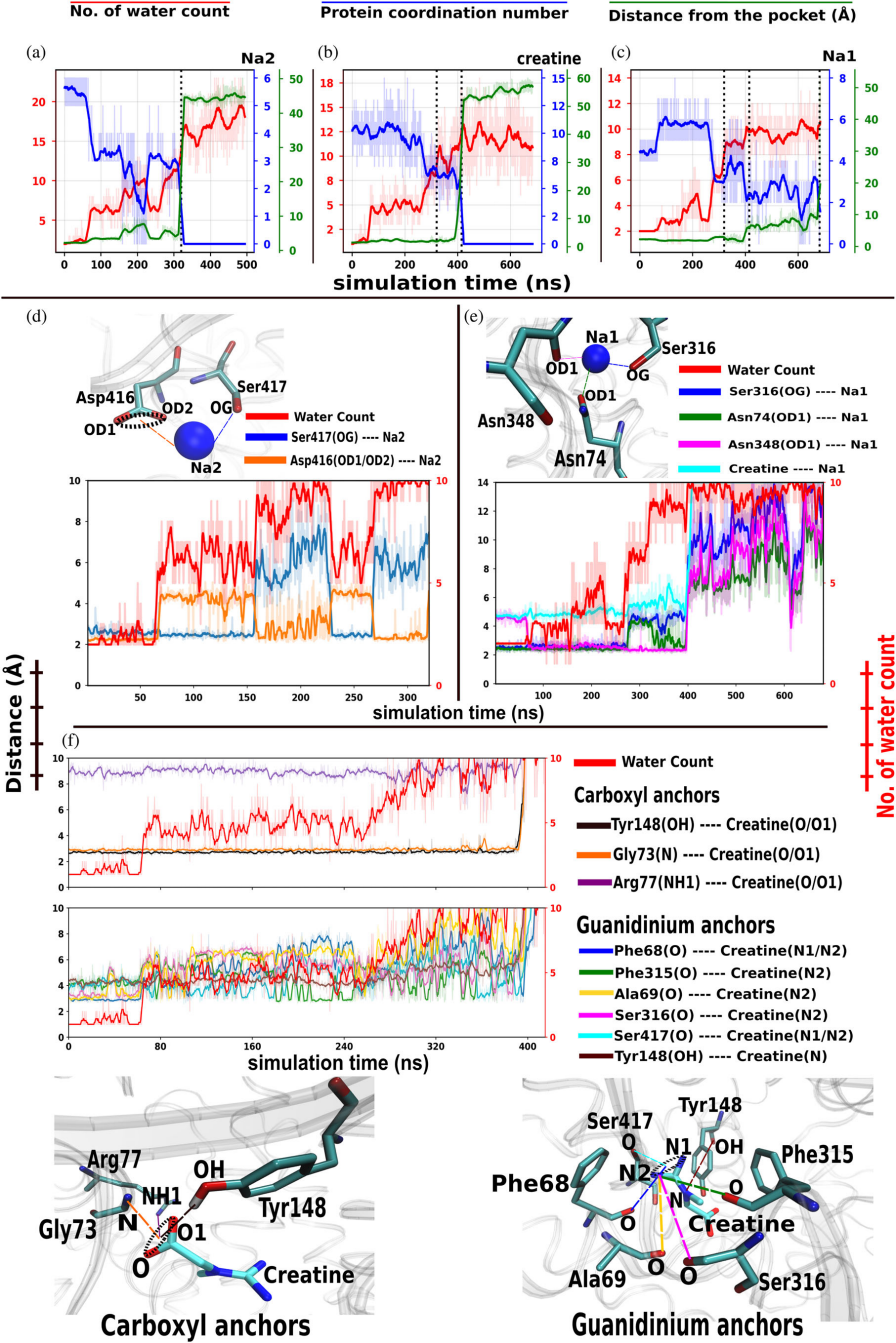
Local hydration, protein coordination, and anchor‐distance analysis for Na2, creatine, and Na1 during intracellular release at 5 pN for a representative replica (replica 1). (a–c) Time series for Na2 (a), creatine (b), and Na1 (c). For each species, the number of water molecules within 3.5 Å (red), the number of nearby protein polar donor atoms (O, N, S) within 3.5 Å (blue), and the center‐of‐mass (COM) distance between the species and residues within 3.5 Å of the species (green) are plotted over simulation time. Dotted vertical lines mark the release times reported in Figure 2 and Table S1 (Data S1). Analyses for all four independent replicas are provided in Figure S5 (Data S1). (d) Hydration and anchor‐distance analysis for Na2. Distances are shown for Ser417(OG)‐Na2 (blue) and Asp416(OD1/OD2)‐Na2 (orange), alongside the number of water molecules within 3.5 Å of Na2 (red). For Asp416, the plotted value represents the minimum OD1/OD2‐Na2 distance at each frame. (e) Hydration and anchor‐distance analysis for Na1. Distances are shown for Ser316(OG)‐Na1 (blue), Asn74(OD1)‐Na1 (green), Asn348(OD1)‐Na1 (magenta), and creatine‐Na1 (cyan), along with the number of water molecules within 3.5 Å of Na1 (red). (f) Hydration and anchor‐distance analysis for creatine. Anchor distances are grouped into carboxylate‐side contacts (Tyr148(OH)‐creatine(O/O1), Gly73(N)‐creatine(O/O1), Arg77(NH1)‐creatine(O/O1)) and guanidinium‐side contacts (Phe68(O)‐creatine(N1/N2), Phe315(O)‐creatine(N2), Ala69(O)‐creatine(N2), Ser316(O)‐creatine(N2), Ser417(O)‐creatine(N1/N2), Tyr148(OH)‐creatine(N)). Distances involving equivalent atoms (OD1/OD2, O/O1, or N1/N2) represent the minimum value per frame. Distances are shown on the left y‐axis (Å), water counts on the right y‐axis (red), and the shared x‐axis corresponds to simulation time (ns). The complete list of anchor pairs is provided in Table S2 (Data S1); individual replica plots are shown in Figures S6–S8.

Next, we examined the changes in the local binding environment, which could help explain the delayed, stepwise release observed earlier. During the release stage, CRT is already in an inward‐open‐like conformation with a constant steering force applied to creatine and Na ions; therefore, one might expect rapid escape once a cytosolic pathway opens. However, Na2, creatine, and Na1 remain stably bound in their pockets for extended periods, and successive release events are separated by significant time gaps. To investigate this behavior, we monitored for each species the number of water molecules within 3.5 Å (red), the number of nearby protein polar donor atoms within 3.5 Å (blue; O, N, S), and the center of mass (COM) distance between the species and nearby residues (green) (Figure 3a–c; Figure S5, Data S1). Across all species and replicas, release consistently follows a pattern: while the species stays in the pocket (low distance), the local environment becomes increasingly hydrated, and protein coordination decreases. These changes occur before the sharp increase in distance that marks the release, indicating hydration develops within the pocket prior to creatine and ions escaping into the bulk solvent.

For Na2, the bound state is characterized by low hydration (~2–3 waters) and strong protein coordination (~5–6). As the simulation progresses, hydration increases and coordination decreases while the ion stays in the pocket. Just before release, transient fluctuations occur, during which coordination temporarily increases as stabilizing contacts are briefly re‐established. Ultimately, hydration rises to ~10–15 waters, and coordination decreases to ~1–2, followed by a sharp increase in distance that marks Na2 release. Creatine shows a similar relationship between hydration and loss of coordination. While creatine remains bound, the local water count increases from ~2 to ~10–15 near release, and protein coordination decreases from ~10 to ~5. Short‐lived fluctuations again appear before release. Since creatine release follows Na2 release, Na2 exit may create additional space for water to enter, coinciding with continued wetting and weakening of creatine‐protein interactions before escape. For Na1, the same pattern occurs over a longer timescale. Na1 stays bound longer than Na2 and creatine, with its release preceded by increased hydration and decreased coordination, typically involving ~1–2 residues in coordination before release. Hydration increases while Na1 remains near the pocket; a sharp increase in distance then marks its release. Overall, these observations suggest that gradual wetting and weakening of protein coordination happen before the release of all transported species.

To identify which stabilizing interactions are disrupted during hydration, we next examined anchor residues that maintain the tightly bound state (Section 4.6). Specifically, we analyzed the distances between creatine or ion atoms and the coordinating protein atoms that form anchoring interactions (Table S2, Data S1). Here, we focus on the strongest stabilizing contacts defining the bound state before release. Anchor residues for creatine were selected from stage 2 of the contact analysis (Section 2.5), where substrate‐protein interactions are most compact, whereas for ions, only the nearby polar atoms were selected based on visual inspection.

At the Na2 binding site, the ion is primarily anchored by Ser417(OG) and Asp416(OD1/OD2), with both distances remaining short (~2–3 Å) in the bound state (Figure 3d; Figure S6 and Table S2, Data S1). As hydration increases, the Asp416‐Na2 distance first increases by ~2–3 Å while the Ser417‐Na2 distance remains stable. With further hydration, the Ser417‐Na2 distance increases by ~3–4 Å while the Asp416 distance returns toward its initial value. This continuous reversible redistribution between anchors indicates that Na2 shifts between alternative stabilizing interactions as hydration progresses. With continued water penetration, Na2 becomes surrounded by water molecules and is eventually released into the cytosol.

Creatine exhibits a related but distinct behavior. Its anchoring interactions were grouped into contacts involving the carboxylate moiety (O/O1) and the guanidinium moiety (N, N1, N2) (Figures 3f and S7 and Table S2, Data S1). Distances between the carboxyl oxygen and Tyr148(OH), Gly73(N), and Arg77(NH1) remain short and stable throughout most of the bound period, only increasing significantly at the moment of release. In contrast, distances involving the guanidinium group, especially Phe68(O) and Ala69(O), and to a lesser extent Phe315(O), Ser316(O), Ser417(O), and Tyr148(OH), fluctuate and grow as hydration increases. These changes suggest a progressive weakening of interactions on the intracellular side of the pocket. Once hydration becomes sufficiently high to destabilize even the stable carboxyl‐side interactions, these contacts are abruptly lost, and creatine is rapidly released into the cytosol.

Within the Na1 binding site, anchoring interactions involve Ser316(OG), Asn74(OD1), and Asn348(OD1) (Figures 3e and S8 and Table S2, Data S1). During early hydration, these distances remain relatively stable, with Ser316(OG)‐Na1 and Asn74(OD1)‐Na1 near ~2–3 Å and Asn348(OD1)‐Na1 near ~4–5 Å. Large fluctuations only appear after creatine release. Once creatine exits the pocket, Na1 anchor distances increase and fluctuate strongly, suggesting that Na1 leaves its primary binding position but does not immediately escape. Instead, Na1 samples an alternative hydrated region near the former Na2 site before its final release (Video S1). Immediately before exit, distances to all anchor residues increase sharply, leading to Na1 release into the cytosol. Although release timing varies between replicas, the same sequence, tight anchoring, progressive hydration, redistribution of anchoring interactions, and final separation is consistently observed across all the replicas for all the species (Figures S6–S8, Data S1).

Together, these analyses show that intracellular release proceeds through progressive wetting of the binding pocket, weakening of stabilizing contacts, and sequential disruption of anchoring interactions for Na2, creatine, and Na1. These observations support a water‐assisted release mechanism in which local hydration precedes and facilitates disengagement of substrate and ions, even when the transporter is already in an inward‐open‐like conformation and a constant driving force is applied. The arrival, progressive hydration of the binding pocket, and the sequential release of all transported species are shown in Video S1.

### 2.4 Electrostatic energy redistribution further assists the release

**FIGURE 4:**
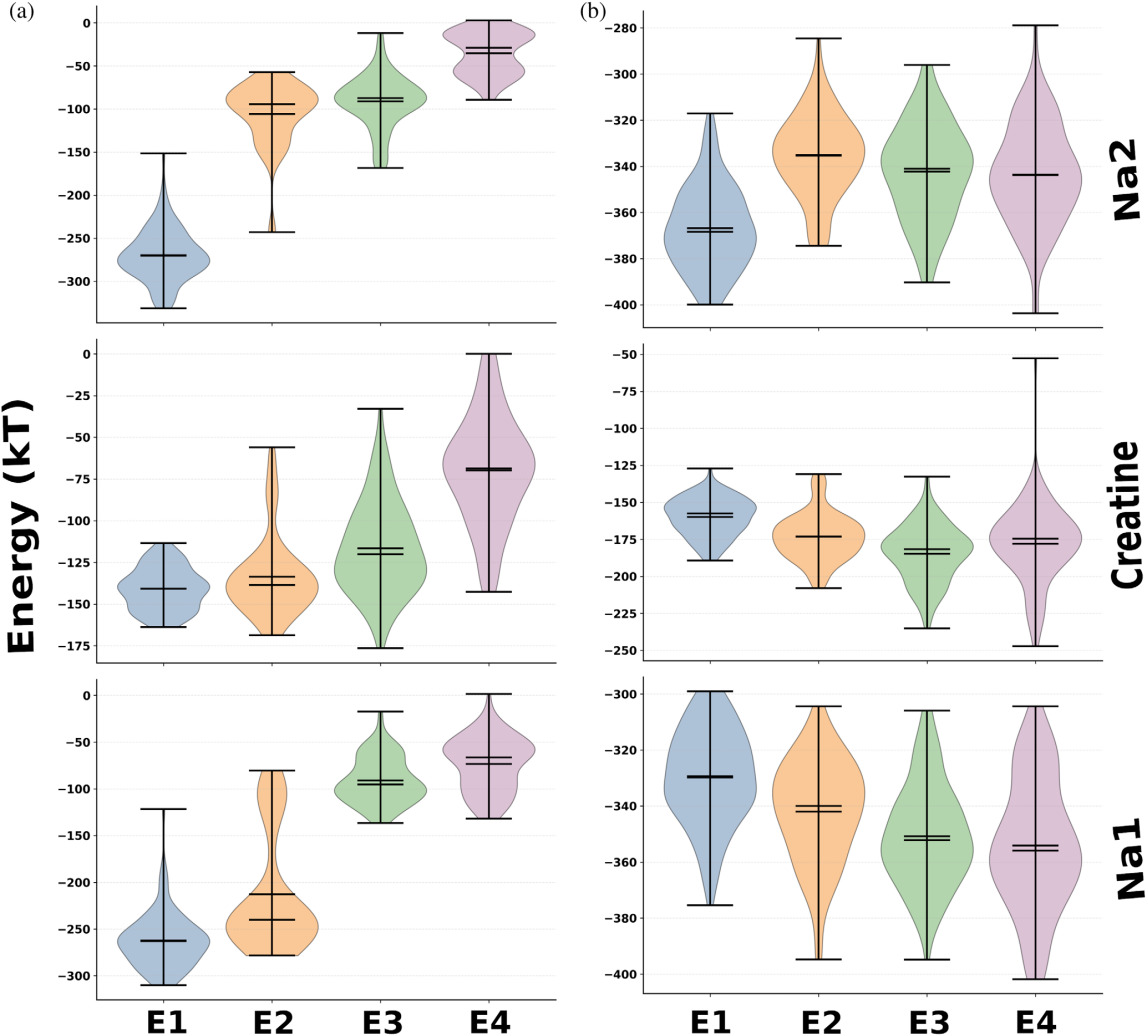
Ensemble‐resolved electrostatic interaction energies for Na2, creatine, and Na1 protein‐lipid environment, with bulk (implicit) water (a), and in the protein‐lipid‐explicit water molecules (b). Violin plots show the distributions of DelPhi‐calculated electrostatic interaction energies (kT) for Na2 (top), creatine (middle), and Na1 (bottom) across four ensembles: tightly bound (E1), bound intermediate (E2), hydrating (E3), and released (E4). Each violin represents the pooled energy distribution across all four replicas for the corresponding ensemble. Ensemble definitions based on hydration and anchor engagement are provided in Table S3 of Data S1.

As established above, release occurs via a water‐assisted mechanism; however, because creatine is a dipole and the ions are charged species, strong electrostatic interactions are likely to stabilize them within their binding sites. Therefore, release requires weakening or redistribution of these interactions. To investigate this, we computed electrostatic interaction energies (Section 4.7) and analyzed them across hydration‐ and anchor‐defined ensembles representing successive stages of destabilization (Table S3, Data S1). Release trajectories were grouped into discrete ensembles based on local hydration and anchor engagement, enabling interpretation of energetic changes in terms of physical release progression rather than simulation time, while preserving the overall temporal order of release. Electrostatic energies were evaluated in both the protein‐lipid environment, with bulk (implicit) water (Figure 4a), and in the protein‐lipid‐explicit water molecules (Figure 4b). This provided an energetic description of electrostatic interactions during release, including the role of water molecules entering the pocket and the sequential release of Na2, creatine, and Na1.

Within the protein‐lipid environment with bulk (implicit) water (Figure 4a), Na2 exhibits strong electrostatic stabilization in the tightly bound ensemble (E1), with interaction energies around −260 to −300 kT, consistent with compact coordination by Asp416 and Ser417 and minimal hydration. A significant energetic change occurs upon transition to E2, where energies become much less favorable (−80 to −120 kT), indicating a weakening of protein‐mediated stabilization as hydration begins. Energies in E2 and E3 remain within a similar range, reflecting reversible fluctuations between Asp416 and Ser417 observed in the anchor‐distance analysis, with Na2 sampling partially stabilized conformations in a hydrated environment rather than immediately escaping. Upon release (E4), energies approach near‐neutral values (−20 to −60 kT), confirming the loss of electrostatic confinement by the protein. Overall, this corresponds to a large decrease in protein‐mediated stabilization from the tightly bound state to the released state, with a consistent monotonic reduction across the E1 to E4 progression. Thus, from the point of view of electrostatic interactions, the most favorable state for Na2 is the bound state in E1 conformation, and, without external support, Na2 will stay bound in the pocket. However, considering explicit water molecules, especially the water molecules that entered the pocket, makes the energy difference much less pronounced. Thus, as protein electrostatic stabilization decreases across the ensembles, water stabilization increases, indicating a gradual shift in the dominant electrostatic interaction from protein anchors to the surrounding solvent, leading to Na2 release.

Creatine shows a smaller energetic response reflecting the asymmetric behavior of its carboxyl and guanidinium anchors. In the protein‐lipid environment (Figure 4a), the tightly bound ensemble (E1) has energies around −140 to −160 kT. Early hydration causes only a small energetic change (−135 to −150 kT), consistent with the preservation of strong carboxyl‐side electrostatics despite partial weakening of guanidinium‐side interactions. A more noticeable reduction in energy appears in the hydrating ensemble (E3), probably marking the Na2 release, where values shift toward −120 to −140 kT as guanidinium‐side fluctuations increase, while carboxyl anchoring remains intact. In the released ensemble (E4), energies increase further, from −60 to −90 kT, indicating disruption of stabilizing carboxyl‐side interactions and increased solvent exposure. When explicit waters are included (Figure 4b), the energetic order reverses, with solvated creatine in E4 becoming the most favorable (−170 to −190 kT), indicating that release occurs when solvent stabilization overcomes remaining protein electrostatic interactions.

Na1 shows a delayed energetic response consistent with its release following creatine release. In the protein‐lipid environment (Figure 4a), the tightly bound ensemble (E1) exhibits strong stabilization near −250 to −290 kT. The transition to E2 results in only moderate weakening (−200 to −260 kT), indicating that Na1 remains largely coordinated during early hydration. A more pronounced energetic shift occurs in the hydrating ensemble, where energies increase toward −90 to −130 kT, aligning with displacement from the primary binding region after creatine release. In the released ensemble (E4), energies increase further, from −40 to −80 kT, confirming the loss of protein electrostatic confinement. When waters are explicitly included (Figure 4b), Na1 remains strongly stabilized (−320 to −360 kT), while solvent interactions grow during hydration, making E4 the most favorable state. This delayed weakening of protein stabilization supports the sequential mechanism in which Na1 release depends on prior creatine release and proceeds through hydration‐assisted relocation before final escape.

Across Na2, creatine, and Na1, a consistent energetic principle appears. Intracellular release is governed not by a sudden loss of electrostatic stabilization but by a redistribution of stabilization from protein coordination to solvent interactions as hydration increases. Protein‐lipid electrostatics gradually weaken from bound to released states, while solvent electrostatics stay stabilizing, allowing release once protein confinement is sufficiently diminished. This energy transfer explains the sequential release of Na2, creatine, and Na1.

### 2.5 Contact residues along the full creatine transport pathway

**FIGURE 5:**
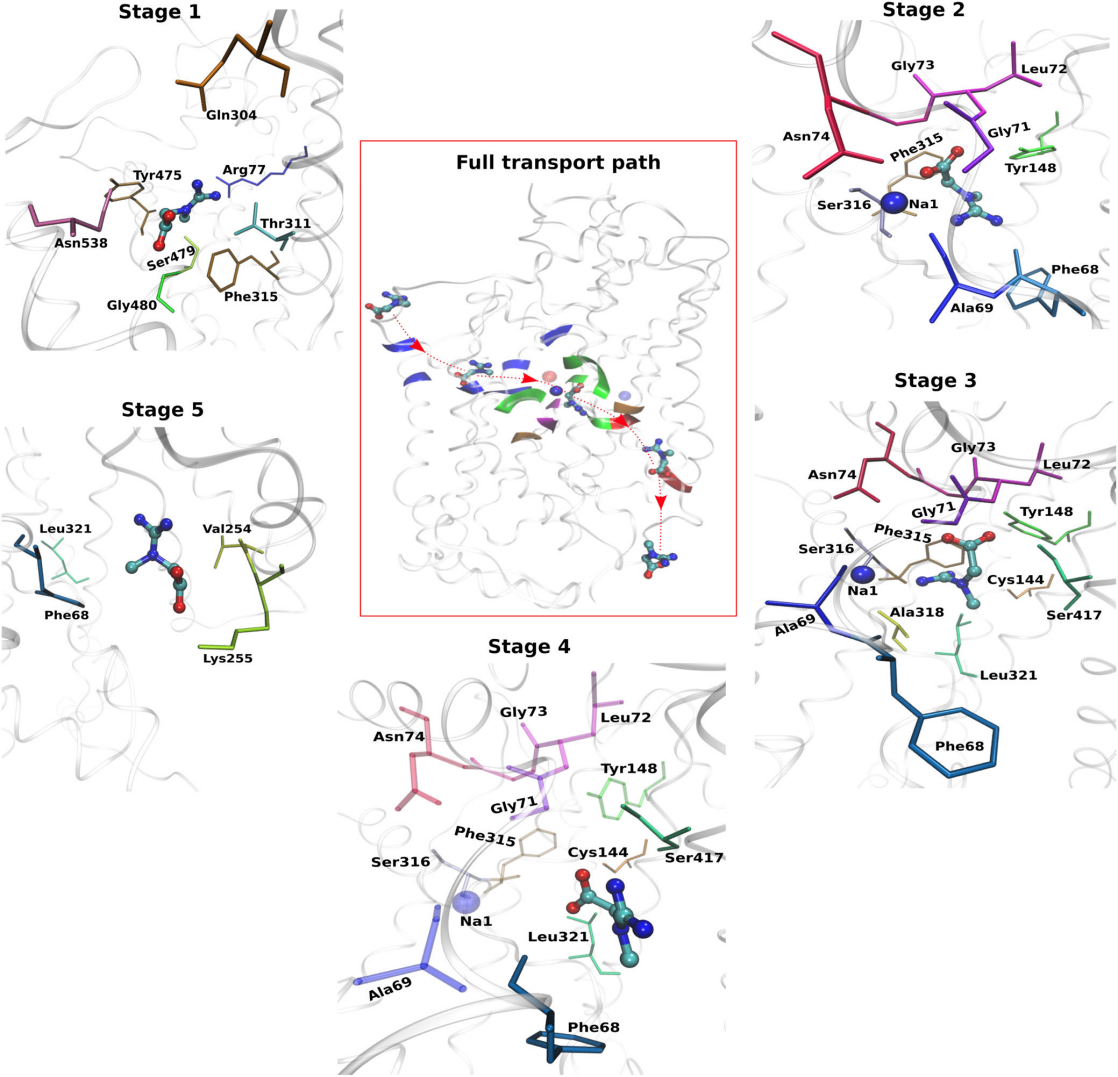
Stage‐wise residues interacting with creatine along the complete transport pathway. Representative structural snapshots illustrating creatine interactions with surrounding residues during five stages of transport. The central panel shows the overall trajectory of creatine as it passes through the transporter, with a red arrow indicating the pathway. The surrounding panels show the residues at individual stages. (Stage 1) Creatine entry from the extracellular vestibule toward the primary binding site (S1). (Stage 2) S1‐bound configuration associated with outward occlusion. (Stage 3) S1‐bound state during transition toward inward‐open conformations. (Stage 4) Destabilization of S1 pocket interactions preceding release. (Stage 5) Creatine progression along the intracellular exit pathway. Residues shown correspond to the most representative creatine‐contact residues at each stage, while the complete lists of contacts are provided in Tables S1–S5 (Data S2). In stage 4, selected residues are partially faded to indicate weakening interactions preceding release. Protein residues are shown as sticks and labeled; creatine is shown in stick representation, and Na1 is shown as a blue sphere whenever it contributes to the interaction.

To identify residues involved in creatine transport, we analyzed residue‐level creatine‐protein contacts across five stages (Figure 5). Stage 1 represents the transport of creatine from its EC placement to its first arrival at the primary binding site (S1). Stage 2 corresponds to the S1‐bound state associated with outward occlusion. Stage 3 corresponds to the S1‐bound conformation, as the transporter transitions toward inward‐open conformations via tMD. Stage 4 represents the destabilization of pocket interactions preceding release, and stage 5 describes the progression of creatine along the intracellular exit pathway.

During stage 1 (Figure 5, Table S1, Data S2), creatine interacts with residues lining the EC vestibule and upper transport pathway. Frequently contacted residues include Asn538, Tyr475, Arg77, Ser479, Gly480, Phe315, Thr311, and Ile307, although occupancies vary across replicas. This variability indicates that creatine approaches the S1 pocket through multiple geometrically distinct entry routes rather than a single fixed pathway. Hydrogen bonds occur intermittently and most often involve Asn538, Ser479, and Tyr475, suggesting that polar interactions help orient creatine during entry while van der Waals contacts dominate the interaction pattern and possibly keep creatine along the pathway. Overall, stage 1 represents a flexible exploratory phase preceding formation of a stable binding configuration. Once creatine reaches S1 during stage 2 (Figure 5, Table S2, Data S2), the contact residue group becomes markedly more compact. High‐occupancy contacts cluster around core S1 residues including Tyr148, Phe315, Gly73, Phe68, Ala69, Gly71, Leu72, Asn74, and Ser316, indicating stabilization of a well‐defined bound conformation. Persistent hydrogen bonds involve Tyr148, Gly73, Phe68, and Ser316, while additional contacts with Ala318, Cys144, and Ser417 appear toward the intracellular side of the pocket. At this stage, creatine also shows robust coupling to the Na1 ion. In the absence of creatine in the pocket, Na1 maintains interactions with surrounding residues and the conserved chloride ion, consistent with a stable outward‐open conformation. Upon creatine binding at S1, Na1 reorganizes its coordination environment, establishing direct interactions with the substrate, while chloride coupling decreases (Video S1). This reorganization accompanies stabilization of the S1‐bound state and progression toward outward occlusion. In contrast, Na2 does not show comparable involvement, suggesting a limited or no role in pocket stabilization or occlusion under these conditions. Beyond contact occupancies, coordinated local side‐chain rearrangements accompanying creatine transport are also observed. In particular, Phe315 undergoes a pronounced rotameric reorientation as creatine enters the pathway and subsequently binds at S1, remaining stabilized in this orientation during outward occlusion. Gln304 undergoes a transient side‐chain reorientation during stage 1, followed by relaxation once creatine becomes fully bound, consistent with a guiding role during entry. In contrast, Tyr148 shows a more modest side‐chain adjustment upon creatine binding, contributing to stabilization of the S1‐bound conformation.

During stage 3 (Figure 5, Table S3, Data S2), creatine remains tightly bound within the S1 pocket and continues to interact with the same core S1 residues identified in stage 2, indicating the persistence of the ion‐coupled bound state as the transporter shifts toward inward‐open conformations. Simultaneously, interactions with residues aligned toward the IC pathway become more evident, particularly Cys144, Ser417, and Leu321. This suggests that creatine begins to reorganize toward the release pathway while still maintaining the S1‐bound configuration. In stage 4 (Figure 5, Table S4, Data S2), the interaction network becomes less compact and more variable across replicas, consistent with destabilization prior to release. Contacts involving residues such as Phe68, Ser417, Cys144, and Leu321 weaken or are intermittent, whereas interactions with the core S1 residues remain detectable. This indicates that the S1 interaction network loosens rather than fully breaking apart. In line with the hydration analysis described above, this stage coincides with increased pocket wetting, thereby weakening the protein‐creatine interaction network and promoting reorganization of contacts. Finally, during stage 5 (Figure 5, Table S5, Data S2), creatine progresses along the IC pathway and forms transient contacts with residues lining the exit corridor. Some interactions that weakened during stage 4, such as those involving Na1, Leu321, and Phe68, reappear in a replica‐dependent manner as creatine samples alternate positions along the pathway. Meanwhile, new contacts emerge with residues further along the intracellular route, including Lys255 and Val254, indicating a gradual transfer of interactions from the S1 pocket to the intracellular exit corridor. Taken together, the stages 1–5 progression outlines a sequential transport pathway in which creatine enters through a flexible extracellular vestibule, converges on a compact, ion‐coupled S1‐bound state that promotes outward occlusion, and then transitions through a destabilized intermediate before moving along the IC exit pathway (Figure 5 and Video S1).

### 2.6 Dynamic network analysis of long‐range communication during creatine transport

**FIGURE 6:**
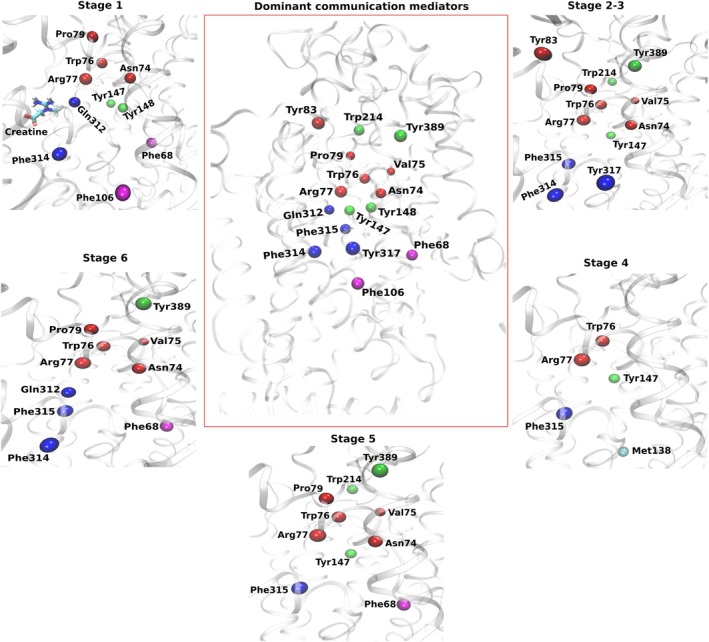
Dynamic network analysis of long‐range communication during creatine transport. Dominant communication mediators identified from bottleneck analysis across the transport cycle. Stage definitions are the same as those used in the contact analysis (stages 1–5), with stage 6 representing the post‐release state after creatine exit. The central panel shows the combined set of dominant communication mediators observed across all the stages, while the surrounding panels display mediators identified for each individual stage. Residues are shown as spheres and labeled. Colors indicate mediator groups: red, TM1 backbone residues; blue, TM6 residues adjacent to the S1 region; green, relay residues linking helices and loops; magenta, aromatic residues toward the intracellular vestibule. Complete mediator lists are provided in Tables S6–S11 (Data S2).

Although the contact analysis above identifies residues that directly interact with creatine along the transport pathway, it does not capture how these local interactions are coordinated across the transporter during the conformational transitions required for alternating access. To characterize this long‐range coupling, we performed stage‐wise dynamic network analysis using the same mechanistic stages defined for the contact analysis (stages 1–5), together with an additional post‐release stage (stage 6) after creatine has completely released (Figure 6). Bottleneck analysis was used to identify residues that recurrently lie along shortest communication paths (Methods, “Dynamic Network Analysis”). Residues with high bottleneck scores are referred to here as dominant communication mediators, representing preferred routing points for coupling among helices during transport.

During the early entry phase (stage 1), dominant communication mediators are broadly distributed across the EC vestibule and the region leading toward the S1 binding pocket (Figure 6; Table S6, Data S2). Recurrent mediators appear along vestibule‐facing regions of TM1 and TM2, including Asn74, Trp76, Arg77, and Pro79, while additional mediators extend toward TM3 residues (Tyr147 and Tyr148) and S1‐adjacent TM6 positions (Gln312 and Phe314). This distribution defines a communication corridor linking extracellular entry to the binding‐site core. At this stage, creatine itself frequently appears as a dominant mediator, consistent with substrate‐assisted communication, as it approaches S1.

Once creatine reaches S1 and the transporter progresses toward outward occlusion and inward open (stages 2–3), the communication network becomes more focused and compact (Figure 6; Tables S7, S8, Data S2). Dominant mediators consolidate along a TM1–TM6 communication backbone linking extracellular gating elements to the S1 binding‐site region. Representative mediators include TM1 residues Asn74, Val75, Trp76, Arg77, and Pro79, together with S1‐adjacent TM6 residues Phe314, Phe315, and Tyr317. Additional relay points extend toward loop regions such as Tyr389, indicating coupling between the TM1/TM6 backbone and EC loops. During this phase, creatine is no longer detected as a dominant mediator, indicating that long‐range communication becomes primarily protein‐encoded once the S1‐bound state is established, probably to initiate the rock‐bundle mechanism. Additional relays also appear through TM3‐associated residues and the connector region near Trp214, which links TM3 and TM4. Notably, Trp214 does not appear as a contact residue in the contact analysis, suggesting that its primary role is to transmit inter‐helix communication rather than directly stabilize the substrate.

A pronounced change occurs during stage 4, corresponding to loosening of creatine from the binding pocket. Dominant communication mediators become substantially less consistent across replicas (Figure 6; Table S9, Data S2), indicating increased heterogeneity in preferred communication routes. Only a small subset of residues remains recurrent across trajectories, while other mediators appear intermittently across helices and loop regions. This reduced cross‐replica consensus suggests that stage 4 represents a structurally heterogeneous regime. Importantly, this behavior parallels the contact analysis, which shows reduced stability and increased variability of several pocket interactions during substrate loosening. Together, these observations indicate that partial destabilization of the bound state is accompanied by redistribution of long‐range communication pathways. As creatine progresses toward the intracellular side (stage 5), a reproducible set of mediators re‐emerges despite continued sparsity of persistent substrate contacts (Figure 6; Table S10, Data S2). Communication again centers on the TM1–TM6 backbone, while additional relay points link this backbone to IC‐facing regions involved in substrate exit. Creatine appears as a dominant mediator in only a subset of trajectories at this stage, indicating that substrate‐assisted communication is weaker than during early entry and that communication is again primarily routed through protein residues. Finally, in the post‐release state (stage 6), dominant mediators become strongly reproducible across replicas (Table S11, Data S2), indicating restoration of a stable communication architecture after substrate exit. Communication again centers on TM1 residues and the S1‐adjacent region of TM6, with additional relay contributions from residues such as Tyr389, while creatine is absent from the network.

Across the full transport cycle, the network analysis reveals a compact set of residues that repeatedly mediate long‐range communication rather than a uniformly distributed network across the transporter. The most consistent mediators are concentrated in TM1 and the S1‐adjacent region of TM6, thereby defining a persistent TM1–TM6 communication backbone that links extracellular gating, substrate binding, and intracellular release. Communication is transmitted to other regions through relay sites, including TM3‐associated residues, the Trp214 connector, and loop positions such as Tyr389. Comparison with the contact analysis shows partial overlap between residues involved in substrate stabilization and those mediating long‐range communication, while positions such as Trp214 and Tyr389 primarily function as relay nodes transmitting inter‐helix coupling.

## 3 DISCUSSION

In this study, we present a continuous molecular description of creatine transport in human CRT (SLC6A8), capturing substrate and ion transport across all stages of the alternating‐access mechanism (Figure S1, Data S1). Using a hybrid simulation approach, we reproduce both the full four conformational states and the experimentally expected sequential release order of Na2, creatine, and Na1. Previous computational studies of CRT have mostly focused on substrate docking or stability within individual conformations, rather than on revealing the entire transport pathway (Clarke et al., 2024; Colas et al., 2020). Additionally, recent cryo‐EM structures have resolved inward‐occluded and inward‐open states (Chen et al., 2025), providing experimental support for the conformational framework investigated here. By combining cf‐sMD, which estimates the electrochemical driving force, with tMD, which directs the transporter along the conformational states, we effectively reconstruct the full transport process.

A key challenge in modeling transporter function is estimating the effective electrochemical driving force operating in vivo. Under physiological conditions, a membrane potential of approximately 60 mV across a ~7 nm membrane corresponds to an average electric field of about 10^7^ V/m, producing forces around 1–2 pN on a monovalent charge. However, this simplified estimate does not consider the heterogeneous dielectric environment of the protein‐membrane system or the effects of ion concentration gradients and transport stoichiometry. Therefore, the steering force used in simulations should be interpreted as an effective driving force rather than the actual physical electric field. Systematic tests identified two forces that are optimal for different stages of transport: a higher force (20 pN) for transporting creatine from the EC vestibule into the S1 binding pocket and for facilitating outward occlusion, and a lower force (5 pN) for releasing creatine and Na^+^ ions into the cytosol. This difference reflects the asymmetry of the transport pathway. During extracellular entry (Figure S9, Data S1), creatine follows a curved vestibular path while the applied force acts along the membrane normal, meaning only part of the force effectively drives motion along the pathway. Entry also occurs into a preorganized ionic environment where Na^+^ and Cl^−^ are already bound, creating steric and electrostatic resistance to substrate insertion. In contrast, intracellular release occurs roughly along the direction of the applied force after tMD has established inward opening, reducing the need for additional large conformational changes. Additionally, the release does not depend solely on force; water also plays a critical role, thereby reducing the effective force required. Overall, the magnitude of the forces was calibrated to reproduce experimental observables (e.g., transport rates, release order) and should not be considered as actual physical forces acting in vivo.

Importantly, cf‐sMD alone does not cause the transition from the outward‐occluded to the inward‐occluded or inward‐open conformations. TM1a bending and rearrangement of intracellular gating residues are not observed within 500 ns when only substrate pulling is applied, indicating that substrate displacement and global conformational transitions are separate processes. This suggests that the transition toward inward opening after occlusion presents a major kinetic barrier in CRT. This interpretation aligns with experimental transport rates (~15 creatine molecules per second), corresponding to timescales of ~10^7^–10^8^ ns per transport cycle (Farr et al., 2022). Once the inward opening is achieved in our simulations, substrate and ion release happen within hundreds of nanoseconds. Overall, these findings show that large‐scale conformational transitions dominate the overall transport timescale in vivo. These slow conformational changes and other effects in vivo are overcome by forced transition from inward‐occluded to inward‐open conformations and applied forces.

A key finding of this study is the direct dynamic observation of an almost strictly ordered intracellular release sequence (Na2 → creatine → Na1). Although this sequence has been proposed by analogy to other NSS transporters, it has not previously been demonstrated dynamically for CRT. Across all successful trajectories at 5 pN, the release order remains invariant, while the total release times vary substantially, suggesting that transport follows a conserved set of mechanistic steps, with kinetics influenced by the lifetimes of intermediate states along the release pathway. Notably, Na1 consistently exhibits the longest release time, suggesting it may be the rate‐limiting step in intracellular release. Importantly, the same 5 pN steering force was applied simultaneously to creatine and both Na^+^ ions, yet the expected release sequence was reliably maintained, showing that the order is driven by the intrinsic transport mechanism under proper external force. During intracellular release, a distinct mechanistic insight emerged: at 5 pN, release occurs in the correct sequence, whereas higher forces (10–20 pN) disrupt this order. This demonstrates that CRT does not operate as a purely mechanical system in which increased force simply accelerates transport. Instead, release depends on a coordinated interplay of conformational relaxation, hydration of the binding pocket, and redistribution of electrostatic stabilization. Larger forces disrupt this coordination and shift the system away from its natural operating regime.

Another key observation is the role of hydration in enabling release. Both creatine and sodium ions remain stably bound even after inward opening and application of force, and dissociation only happens when water molecules progressively penetrate the binding pocket. Hydration, therefore, occurs beforehand and facilitates release rather than resulting from it. As water enters the pocket, it weakens protein coordination and changes the electrostatic environment by competing with coordinating residues. Electrostatic analysis supports this view, showing that the stabilization of creatine and Na^+^ ions redistributes in the binding environment as hydration increases. Release occurs only after sufficient solvent‐mediated weakening of these interactions. Transport is thus governed by the interplay among the electrochemical driving force, protein conformational changes, local hydration dynamics, and shifts in electrostatic energy.

Residue‐level contact and dynamic network analysis further clarify how transport is coordinated. During entry, creatine interacts with a wide range of EC vestibular residues, supporting multiple possible entry pathways. Upon binding in the S1 site, the interaction network becomes more compact, involving residues such as Tyr148, Phe315, Gly73, Ser316, and Asn74. Na1 reorganizes its coordination environment upon substrate binding, stabilizing the occluded state. In contrast, Na2 has a limited role in substrate recognition but acts as an initiator of the release process. Network analysis also shows that communication pathways consolidate along a TM1–TM6 backbone during occlusion, redistribute before release, and re‐establish a stable architecture after substrate exit, consistent with the rocking‐bundle mechanism typical of LeuT‐fold transporter (Forrest & Rudnick, 2009).

Given the strong structural conservation within the SLC6/NSS family, the mechanistic features identified here are likely widely applicable. In particular, the sequential release mechanism, the primary role of TM1 helices in gating, and the coupling between hydration and electrostatic destabilization may serve as general principles of NSS transporter function. The hybrid cf‐sMD/tMD framework introduced here, therefore, offers a transferable approach for studying transport mechanisms in related systems. Additionally, these findings have significant implications for understanding disease‐related mutations in SLC6A8 (van de Kamp et al., 2013). Additionally, the same protocol used here can be extended to study transport in the CRT variants. Pathogenic variants may disrupt anchoring residues or hydration pathways, impair sequential release, trap the transporter in nonfunctional conformations, or alter contacts or network pathways. By providing a continuous mechanistic view of creatine transport, this work lays the foundation for linking molecular‐level disruptions to functional defects and guiding future therapeutic strategies. Indeed, the protocol can be applied to study the effect of pathogenic mutations of the creatine transport and to identify plausible binding patches for small molecules, which binding could mitigate the effect of these mutations. In summary, creatine transport through CRT emerges as a coordinated process governed by the interplay of electrochemical driving forces, protein conformational transitions, and water‐mediated destabilization of binding interactions. By capturing the complete transport cycle and identifying determinants of substrate and ion release, this study establishes a mechanistic framework for CRT function and a generalizable strategy for studying transport within the SLC family.

## 4 METHODS

### 4.1 Structural models and ligand preparation

As mentioned, the creatine transporter adopts four distinct conformations during creatine transport: outward open, outward occluded, inward occluded, and inward open. Although the goal of this investigation is to start from the outward open conformation and sample the remaining conformations as creatine progresses along its pathway, the experimental structures of the inward occluded (PDB: 9KRH) (Chen et al., 2025) and inward open (PDB: 9KR7) (Chen et al., 2025) conformations were obtained from the Protein Data Bank to aid the modeling process. The initial structure, the outward‐open CRT crystal structure, is unavailable; a homology model from previous work was used (Clarke et al., 2024). This outward open model contains two Na^+^ ions (Na1 and Na2) and one Cl^−^ ion at the binding site, with no substrate creatine at the primary binding site S1 (Figure S2, Data S1, hereafter referred to as “CRT pre‐complex”); therefore, we manually placed creatine approximately 36 Å from the primary binding site (S1) along the z‐axis (Figure S2, Data S1) (hereafter called “CRT‐crt unbound‐complex”). The creatine structure was obtained from PubChem (CID: 586) (Kim et al., 2021), preprocessed with OpenBabel (O'Boyle et al., 2011), and parameterized in Antechamber using the GAFF2 force field (Wang et al., 2004) and the ABCG2 charge model (He et al., 2020; Sun et al., 2023).

### 4.2 Membrane system

The bilayer system was built using the PACKMOL‐Memgen package (Schott‐Verdugo & Gohlke, 2019) in AMBER24 (Case et al., 2023; Case et al., 2025). The “CRT‐crt unbound‐complex,” as described above, was embedded in a POPC:cholesterol bilayer at a 3:1 ratio. The bilayer was built with nloop and nloop_all set to 500 and 2000, respectively, and all other parameters set to the default PACKMOL‐Memgen settings. Further solvation and parameter generation were performed using ‘tleap’ within PACKMOL‐Memgen. The system was solvated using the TIP3P (Jorgensen et al., 1983) water model and neutralized with 0.15 M NaCl. Force‐field assignments included ff14SB (Maier et al., 2015) for the protein, Lipid21 (Dickson et al., 2022) for the lipids, and GAFF2 (Wang et al., 2004) for creatine. An initial short energy minimization was also performed in PACKMOL‐Memgen using default settings. The final system consisted of a rectangular box (126 Å × 126 Å × 111 Å) containing the “CRT‐crt unbound‐complex,” 328 POPC and 109 cholesterol molecules, 72 Cl^−^ and 71 Na^+^ ions, and approximately 26,500 water molecules.

### 4.3 Molecular dynamics simulations

Following bilayer construction, a rigorous first‐stage energy minimization was performed in AMBER24 (10,000 steps total: 5000 steepest descents (SD) followed by 5000 conjugate gradient [CG]). This was first performed with gradually reduced positional restraints on the CRT‐crt complex and lipids (100, 50, 25, 10, 5 kcal·mol^−1^·Å^−2^), then on the CRT‐crt complex only (5, 2, 1 kcal·mol^−1^·Å^−2^). From this point onward, we switched to NAMD 3.0.1 (Phillips et al., 2020), in which an initial unrestrained minimization of 10,000 steps was performed. Next, the system was heated from 0 to 300 K in six stages, each increasing the temperature by 50 K over 10 ps in the NVT ensemble, while applying a 25 kcal·mol^−1^·Å^−2^ positional restraint to the “CRT‐crt unbound‐complex” and lipids. Following this, a second minimization (10,000 steps) was performed under the NVE ensemble, with restraints decreased stepwise on the “CRT‐crt unbound‐complex” and lipids (25, 10 kcal·mol^−1^·Å^−2^), then on the “CRT‐crt unbound‐complex” only (5, 2, 1 kcal·mol^−1^·Å^−2^). The system was then equilibrated at 300 K in the NVT ensemble for 5 ns with a 2 fs timestep and a 10 kcal·mol^−1^·Å^−2^ restraint. Up to this point, calculations were performed on the CPU; subsequent runs used the GPU. NVT equilibration was then continued on the GPU, with restraints gradually reduced to 5, 2, and 1 kcal·mol^−1^·Å^−2^, each for 10 ns, using a 2 fs timestep. During all NVT equilibrations, the restraint was applied to the “CRT‐crt unbound‐complex” and lipids. Following this, the system was equilibrated in the NPT ensemble at a target pressure of 1.01325 bar by gradually decreasing restraints stepwise on the “CRT‐crt unbound‐complex” and lipids (10, 5 kcal·mol^−1^·Å^−2^) for 5 ns each, then on the “CRT‐crt unbound‐complex” only (5, 3, 2, 1 kcal·mol^−1^·Å^−2^) for 5, 5, 5, and 10 ns, respectively. The timestep for all of these NPT simulations was 1 fs. A final 50 ns NPT equilibration was conducted with a 2 fs timestep, restraining only the creatine ligand at 1 kcal·mol^−1^·Å^−2^. Equilibration was assessed by analyzing the system's energy profile, and bilayer stabilization was evaluated using the area‐per‐lipid profile. Both metrics stabilized, so we proceeded with the production runs. All production runs used a 2 fs timestep. For all NPT equilibration, the ratio of the unit cell in the x‐y plane was kept constant while allowing fluctuations along all the axes. Observing that the area per lipid after equilibration was stabilized, the production run was carried out by keeping the area of the unit cell in the x‐y plane constant while allowing fluctuations along the z axis, and thus all production runs were carried out in a constant area NPT (NPAT) ensemble.

Across all NAMD stages, temperature was controlled using Langevin dynamics (Grest & Kremer, 1986) with a damping coefficient of 1 ps^−1^ and a target Langevin temperature of 300 K. Pressure was controlled with a Nosé‐Hoover Langevin piston (Feller et al., 1995; Martyna et al., 1994), with piston period, decay, and piston temperature set to 200 fs, 100 fs, and 300 K, respectively. Particle mesh Ewald (PME) (Darden et al., 1993; Essmann et al., 1995) handled long‐range electrostatics with periodic boundary conditions, and nonbonded interactions used a switching function with a 12 Å cutoff, switching at 10 Å, and a 14 Å pairlist distance. For all production runs, trajectories and energies were saved every 20 ps.

### 4.4 Constant force sMD and targeted MD

To account for the electrical gradient driving creatine across CRT, cf‐sMD simulations were performed in NAMD 3.0.1 using the equilibrated “CRT‐crt unbound‐complex.” A constant steering force was applied to the center of mass of creatine and Na+ ions (Na1 and Na2), with all other MD parameters identical to those described above. Targeted MD (tMD) (Schlitter et al., 1993) simulations were also conducted in NAMD 3.0.1 to drive the conformational transition between the specified conformations. In tMD, a subset of atoms is guided toward a final target structure by means of steering forces described by the potential:

$$U_{\mathrm{tMD}}\left(t\right)=\frac{K}{2N}{\left[\mathrm{RMS}\left(t\right)-\mathrm{RM}S^{*}\left(t\right)\right]}^{2}$$

where RMS(t) is the instantaneous best‐fit RMSD, RMS*(t) decreases linearly from its initial to final value, k is the spring constant, and *N* is the number of targeted atoms. The spring constant was k = 200 kcal·mol^−1^·Å^−2^, and the two tMD simulations used target structures corresponding to inward occluded (PDB: 9KRH) and inward open (PDB: 9KR7). Each tMD simulation was run for 50 ns, and all other MD parameters were set as described above. Three to five independent production runs were carried out for each simulation described in this work. Post‐processing and trajectory analyses were performed using CPPTRAJ (Roe & Cheatham III, 2013), VMD (Humphrey et al., 1996; Stone, 1998), and UCSF Chimera (Pettersen et al., 2004).

### 4.5 Contact analysis

The residue‐substrate contact analysis was performed to identify protein residues that interact with creatine and, where applicable, co‐transported ions as they progress through the transporter protein. Contacts were extracted using the “GetContacts tools” (available at https://getcontacts.github.io/). Time‐resolved protein‐creatine contacts were generated with “get_dynamic_contacts.py,” and residue‐level contact occupancies were computed for each stage and replica. Interaction types were defined according to the standard categories implemented in the “GetContacts” framework. For each trajectory segment corresponding to a defined transport stage, contact occupancies were calculated as the fraction of simulation frames in which a given residue made at least one contact with creatine. Stage definitions are described in the Results. Because different stages represent distinct physical regimes of the transport process, stage‐specific occupancy thresholds were applied during analysis. In particular, stages 1, 4, and 5 correspond to short‐lived transition segments, whereas stages 2 and 3 represent comparatively long‐lived bound states. Accordingly, occupancy thresholds were adjusted to account for these differences in temporal sampling and interaction stability. Specifically, van der Waals, hydrogen‐bond, and salt‐bridge contacts were filtered using stage‐dependent occupancy cutoffs of 20/5/3% (stage 1), 50/10/5% (stages 2–3), and 20/10/10% (stages 4–5). Residues exceeding the corresponding cutoff in at least two independent replicas were retained for reporting in the results. Complete contact frequency tables for all stages across all replicas are provided in Data S2 (Tables S1–S5). Because a steering force of 20 pN was identified as the optimal force for transporting creatine to S1 and inducing outward occlusion, the analysis for stages 1 and 2 focuses primarily on this condition and was performed across three independent replicas. For the release process, 5 pN was identified as the optimal force; accordingly, the analysis of stages 3, 4, and 5 is based on four independent replicas generated under this condition.

### 4.6 Anchor residues identification and distance‐based analysis

To examine how specific stabilizing interactions evolve during intracellular release, a targeted, anchor‐based refinement of the residue‐substrate contacts identified above was performed. This step focuses on a reduced set of persistent, chemically strong interactions. For creatine, the contacts were filtered to include only those involving the carboxylate (O, O1) and guanidinium (N, N1, N2) functional groups. The candidate contacts were then ranked by occupancy and interaction type, and a compact set of anchor interactions was selected by retaining residues that were both frequently observed in the bound state and chemically interpretable as hydrogen bonds or salt bridges, yielding a representative set of stabilizing interactions for creatine binding. For Na2 and Na1, anchor interactions were selected by inspecting the contact analysis output, focusing on persistent polar or charged coordinating residues known to stabilize ions in their binding pockets. Anchors were selected based on their consistent presence during the bound phase (stage 2). The final anchor sets for creatine, Na2, and Na1 are listed in Table S2 of Data S1. When equivalent atoms were present (e.g., creatine O/O1 or N1/N2, or equivalent carboxylate oxygens coordinating Na2), the minimum distance among equivalent atom pairs was used to define a single flip‐invariant anchor distance. An anchor was considered engaged when the interatomic distance was ≤3.5 Å, consistent with hydrogen‐bonding or close coordination interactions.

### 4.7 Ensemble identification for electrostatic energy calculation using DelPhi

To characterize the mechanistic progression of intracellular release for Na2, creatine, and Na1, release trajectories were divided into discrete ensembles based on local hydration and anchor engagement derived from the anchor residue analysis above. For each species, the local hydration number (N_w) was defined as the number of water molecules within 3.5 Å, and the anchor engagement number (N_a) was defined as the number of anchor contacts satisfying the same distance criterion above. Using these two metrics, four ensembles representing progressive destabilization were defined: the tightly bound ensemble (E1), the bound intermediate ensemble (E2), the hydrating ensemble (E3), and the released ensemble (E4). These ensembles were selected to reflect progressive weakening of stabilizing protein‐substrate interactions accompanied by increasing hydration, with numerical thresholds adapted to the coordination environment of each species. The exact criteria used for Na2, creatine, and Na1 are summarized in Table S3 of Data S1. For each species and stage, all frames satisfying the corresponding criteria were identified independently in each replica. To obtain representative snapshots for electrostatic analysis, up to 25 evenly spaced frames per stage were selected from each replica. All selected frames corresponding to the same ensemble from individual replicas were then combined into a single pooled set for that ensemble. Thus, frames from four replicas were merged stage‐wise, yielding four final ensemble datasets for subsequent analyses. All reported electrostatic results (Figure 4) therefore reflect this replica‐merged ensemble representation.

Following ensemble classification and representative frame selection, electrostatic interaction energies were computed using the FRC module of DelPhi (Li et al., 2012; Panday et al., 2025). For each species (Na2, creatine, and Na1), interaction energies were computed by designating the species of interest as the target and treating the remaining components as the source. Calculations were performed in two distinct dielectric environments. In the first environment, the “protein‐lipid‐implicit solvent” environment, the source comprised an explicit protein, an explicit lipid bilayer, and an implicit water phase (Figure 4a). In the second environment, the “protein‐lipid‐explicit solvent,” the source comprised the explicit protein, explicit lipid bilayer, and explicit water molecules (Figure 4b). Electrostatic potentials were solved on a cuboidal grid with a scaling factor of 2.0 and a 50 Å margin, with the solute dielectric constant set to 1.0 and dipolar boundary conditions applied. Convergence was achieved with an initial iteration limit of 5000 and a maximum potential change threshold of 5 × 10^−4^. Target‐source interaction energies were obtained by setting the target charge to zero.

### 4.8 Dynamic network analysis

Dynamic network analysis was performed using the DyNetAn Python package, following the workflow described in the original method paper (Melo et al., 2020). Networks were constructed in a stage‐resolved manner using the same stage definitions as in the contact frequency analysis, with one additional stage (stage 6) corresponding to the stage when creatine is completely released. The simulated system was represented as a residue interaction network, in which each protein residue was modeled as a node defined by its Cα atom, while creatine and each co‐transported ion were represented by single nodes. An edge was assigned between two nodes if the minimum distance between any pair of heavy atoms belonging to the two nodes was within 4.5 Å for at least 10% of frames used within that stage. Contacts within the same residue and between consecutive residues were excluded before network calculations. Generalized correlations between node motions were computed and used to construct correlation‐weighted networks and optimal communication paths, with shortest paths computed using the Floyd‐Warshall algorithm (Melo et al., 2020). Edge betweenness centrality was computed on the correlation‐weighted network, and node‐level bottleneck scores were derived by summing the betweenness of edges incident to each node to identify residues mediating long‐range coupling. In the Results (Section 2.6), residues with high bottleneck scores are referred to as dominant communication mediators for ease of reference.

## AUTHOR CONTRIBUTIONS

**Pitambar Poudel:** Conceptualization; data curation; formal analysis; visualization; writing – original draft; methodology; investigation. **Emil Alexov:** Conceptualization; supervision; project administration. **Shailesh Kumar Panday:** Methodology; investigation.

## CONFLICT OF INTEREST STATEMENT

The authors declare no competing interests. The author(s) used OpenAI ChatGPT (GOT‐5.2) solely for language editing and clarity improvement. The author(s) reviewed, revised, and approved the final manuscript and take full responsibility for its content.

## Supporting information


**Data S1.** Supporting information.
**FIGURE S1.** Cartoon model illustrating creatine transport in CRT based on the alternating‐access mechanism. S1 indicates the creatine‐binding site. Na1 and Na2 are binding sites for Na1 and Na2 ions.
**FIGURE S2**. Manual placement of creatine in the extracellular vestibule, ~36 Å from the S1 binding pocket in the outward open conformation of CRT. The protein is shown as a white cartoon, creatine as orange van der Waals spheres, and ions as spheres (red: Cl^−^; blue: Na^+^). The red arrow indicates the intended path for creatine.
**FIGURE S3.** Creatine entry into the S1 site and extracellular (TM1b/TM6a‐TM9(up)) gating dynamics under different steering forces. This pertains to results obtained when the steering force was applied only to the creatine molecule. (A‐C) COM distance between creatine and S1‐site residues at 5 pN (A), 10 pN (B), and 20 pN (C), shown for three replicas (black, blue, red). The solid line indicates the corresponding distance in the outward‐open homology model used as the starting structure; the dashed line indicates the distance in the inward‐occluded crystal structure (PDB 9KRH) (Hediger et al., 2004) shown for reference. At 5 pN, creatine does not enter the pocket; at 10 and 20 pN, it reaches S1 in all replicas and remains stably bound for the remainder of the simulation. (D, E) TM1b‐TM9(up) COM distance under 10 pN (D) and 20 pN (E). The dashed line indicates the corresponding distance in the outward‐open homology model. A reduction in this distance reflects extracellular‐gate closure. At 20 pN, outward occlusion is observed in all replicas, with replica 3 (red) showing the most pronounced and sustained closure. (F, G) TM6a‐TM9(up) COM distance under 10 pN (F) and 20 pN (G). The dashed line indicates the corresponding distance in the outward‐open homology model. TM6a shows limited early gating motion; modest reductions in replica 3 at 20 pN complement the TM1b‐TM9(up) closure seen in panel E. Overall, TM6a exhibits less early movement relative to TM9(up) than TM1b does. The x‐axis and y‐axis are shared across all panels and represent simulation time (ns) and distance (Å), respectively. Additionally, the plotted traces correspond to locally averaged distances calculated over a 1‐ns sliding window.
**FIGURE S4.** TM1a conformational changes across representative hybrid cf‐sMD and tMD simulations. Creatine is shown as orange van der Waals spheres, and ions as spheres (red, Cl^−^; blue, Na^+^). Results are shown for a representative replica. (Trial 1) TM1a conformational changes during cf‐sMD with steering forces applied simultaneously to creatine and sodium ions. Structures correspond to the end of cf‐sMD (blue), the fully equilibrated outward‐open homology model used as the starting structure (red), and the inward‐occluded crystal structure (green; PDB 9KRH). The observed behavior is comparable to that obtained when steering creatine alone (Figure 2, F, main text). (Trial 2) TM1a conformations from different stages of the simulation: the outward‐occluded starting conformation at 150 ns before tMD (red), the structure at the end of 50 ns tMD toward the inward‐occluded state (green), the conformation after removal of tMD restraints during subsequent cf‐sMD (blue), and the inward‐occluded crystal structure (magenta; PDB 9KRH). TM1a bends toward the inward‐occluded orientation during tMD but relaxes toward the pre‐tMD conformation after restraint release. (Trial 3, left) TM1a conformations from the outward‐occluded state at 150 ns before tMD (red), the end of second‐stage tMD toward the inward‐open state (green), the post‐tMD cf‐sMD conformation (blue), and the inward‐open crystal structure (magenta; PDB 9KR7). TM1a bends toward an inward‐open orientation during tMD but partially relaxes afterward. (Trial 3, right) Superposition of Phe68 with the same color scheme, revealing a noticeable shift in side‐chain orientation.
**FIGURE S5.** Local hydration and protein coordination changes analysis for intracellular release events at 5 pN. Time series are shown for Na2 (left column), creatine (middle column), and Na1 (right column) across Replicas 1–4 (rows). For each species, the number of water molecules within 3.5 Å (red), the number of nearby protein polar donor atoms (O, N, S) within 3.5 Å (blue), and the COM distance between the species and residues within 3.5 Å of the species (green) are plotted as a function of simulation time. Dotted vertical lines mark the corresponding release times reported in Figure 2 in the main article and Table S1.
**FIGURE S6.** Hydration and anchor‐distance analysis for Na2 across all four replicas. The blue sphere represents Na2. Distances are shown for Ser417(OG)‐Na2 (blue) and Asp416(OD1/OD2)‐Na2 (orange), along with the number of water molecules within 3.5 Å of Na2 (red). For Asp416, the plotted value is the minimum of the OD1‐Na2 and OD2‐Na2 distances at each frame. All panels share a common x‐axis of simulation time (ns). Distances are reported on the left y‐axis (Å, black), and water counts on the right y‐axis (red). The complete list of protein‐Na1 anchor pairs is provided in Table S2.
**FIGURE S7.** Hydration and anchor‐distance analysis for creatine across all four replicas. Anchor‐distance time series are separated into carboxylate‐side (upper) and guanidinium‐side (lower) contacts, each plotted with the number of water molecules within 3.5 Å of creatine (red). Carboxylate anchors (upper): Tyr148(OH)‐creatine(O/O1) (black), Gly73(N)‐creatine(O/O1) (orange), and Arg77(NH1)‐creatine(O/O1) (purple). Guanidinium anchors (lower): Phe68(O)‐creatine(N1/N2) (blue), Phe315(O)‐creatine(N2) (green), Ala69(O)‐creatine(N2) (gold), Ser316(O)‐creatine(N2) (magenta), Ser417(O)‐creatine(N1/N2) (cyan), and Tyr148(OH)‐creatine(N) (brown). For anchors involving equivalent atoms (e.g., O/O1 or N1/N2), the plotted distance is the minimum at each frame. All panels share a common x‐axis of simulation time (ns). Distances are reported on the left y‐axis (Å, black), and water counts on the right y‐axis (red). The complete list of protein‐creatine anchor pairs is also provided in Table S2.
**FIGURE S8.** Hydration and anchor‐distance analysis for Na1, shown across all four replicas. The blue sphere represents Na1. Distances are shown for Ser316(OG)‐Na1 (blue), Asn74(OD1)‐Na1 (green), Asn348(OD1)‐Na1 (magenta), and creatine‐Na1 (cyan), along with the number of water molecules within 3.5 Å of Na1 (red). All panels share a common x‐axis of simulation time (ns). Distances are reported on the left y‐axis (Å, black), and water counts on the right y‐axis (red). The complete list of protein‐Na1 anchor pairs is provided in Table S2.
**FIGURE S9.** Force projection along the creatine extracellular entry and intracellular release. A constant steering force is applied to the substrate along the membrane normal (green dashed arrow). During extracellular (EC) entry, creatine follows a curved vestibular pathway toward the S1 binding site (red arrow). Because the applied force is not colinear with the reaction coordinate, only the projected component of the force (*F*_effective_ = *F* cosθ) contributes to motion along the pathway. In contrast, during intracellular (IC) release, substrate movement occurs approximately along the direction of the applied force (black arrow), reducing the required force magnitude.
**TABLE S1.** Release times (ns) for Na2, creatine, and Na1 across five replicas under constant steering forces of 5, 10, and 20 pN. Each cell reports release times in the order Na2/creatine/Na1. “Not out” indicates that the corresponding species did not release within the simulated time. Simulations were terminated upon violation of the expected sequential release order, and trajectories with no release events were terminated at ~700 ns. All release times reported in Table S1 are measured from the start of the tMD protocol (*t* = 0 at tMD initiation), that is, after the 150 ns outward occluded starting structure was selected.
**TABLE S2.** Protein‐substrate anchor contacts used for distance and stage analyses. Protein‐substrate anchor contact pairs. Creatine anchors are grouped according to interactions with the carboxylate moiety (O/O1) or the guanidinium moiety (N, N1, N2). For anchors involving equivalent atoms, the reported distance corresponds to the minimum of the listed atom pairs (e.g., O/O1, OD1/OD2, or N1/N2). Anchor sets consisted of 2 contacts for Na2, 8–10 contacts for creatine, and 3 contacts for Na1.
**TABLE S3.** Ensemble definitions based on hydration and anchor engagement. Criteria used to classify simulation frames into different ensemble stages for Na2, creatine, and Na1 during the release simulations. N_w is the number of water molecules within 3.5 Å of the species. N_a is the number of engaged protein‐substrate/ions anchor contacts if the distance between them is <= 3.5 Å.

**Data S2.** List of residues contacting creatine along with the type of interaction and the occupancy.

**Video S1:** Video of the creatine being transported by the creatine transporter.

## Data Availability

All data required to evaluate the conclusions are provided in the paper and/or in Data S1, Data S2, and Video S1. The simulated trajectory files are available from the corresponding author upon request. The protein structures were obtained from the Protein Data Bank. The creatine molecule was obtained from the public library cited within the article. The programs used for preparation, simulation, and analysis are freely available for download and cited within the article.
