# Supplementary material for: From extracellular entry to intracellular release: A water‐assisted transport cycle for creatine in SLC6A8
Source: Protein Sci. 2026 Jun 18;35(7):e70671. doi: 10.1002/pro.70671 (PMC13279881; doi:10.1002/pro.70671)
Supplement: Supplementary file 1 — Data S1. Supporting information. FIGURE S1. Cartoon model illustrating creatine transport in CRT based on the alternating‐access mechanism. S1 indicates the creatine‐binding site. Na1 and Na2 are binding sites for Na1 and Na2 ions. FIGURE S2. Manual placement of creatine in the extracellular vestibule, ~36 Å from the S1 binding pocket in the outward open conformation of CRT. The protein is shown as a white cartoon, creatine as orange van der Waals spheres, and ions as spheres (red: Cl−; blue: Na+). The red arrow indicates the intended path for creatine. FIGURE S3. Creatine entry into the S1 site and extracellular (TM1b/TM6a‐TM9(up)) gating dynamics under different steering forces. This pertains to results obtained when the steering force was applied only to the creatine molecule. (A‐C) COM distance between creatine and S1‐site residues at 5 pN (A), 10 pN (B), and 20 pN (C), shown for three replicas (black, blue, red). The solid line indicates the corresponding distance in the outward‐open homology model used as the starting structure; the dashed line indicates the distance in the inward‐occluded crystal structure (PDB 9KRH) (Hediger et al., 2004) shown for reference. At 5 pN, creatine does not enter the pocket; at 10 and 20 pN, it reaches S1 in all replicas and remains stably bound for the remainder of the simulation. (D, E) TM1b‐TM9(up) COM distance under 10 pN (D) and 20 pN (E). The dashed line indicates the corresponding distance in the outward‐open homology model. A reduction in this distance reflects extracellular‐gate closure. At 20 pN, outward occlusion is observed in all replicas, with replica 3 (red) showing the most pronounced and sustained closure. (F, G) TM6a‐TM9(up) COM distance under 10 pN (F) and 20 pN (G). The dashed line indicates the corresponding distance in the outward‐open homology model. TM6a shows limited early gating motion; modest reductions in replica 3 at 20 pN complement the TM1b‐TM9(up) closure seen in panel E. Overall, TM6a exhibits les [file PRO-35-e70671-s001.pdf]

SUPPORTING INFORMATION 1

From extracellular entry to intracellular release: A water-assisted transport cycle for creatine in SLC6A8

Pitambar Poudel[1], Shailesh Kumar Panday[1], Emil Alexov[1,2,3*]

[1]Department of Physics & Astronomy, College of Science, Clemson University, Clemson, SC 29634, USA

[2]Medical Biophysics Program, Clemson University, Clemson, SC 29634, USA

[3]Clemson University Center for Human Genetics, Greenwood, SC 29646, USA

***Corresponding author:** Emil Alexov, ealexov@g.clemson.edu, 110 Kinard Laboratory, Clemson, SC 29634, USA

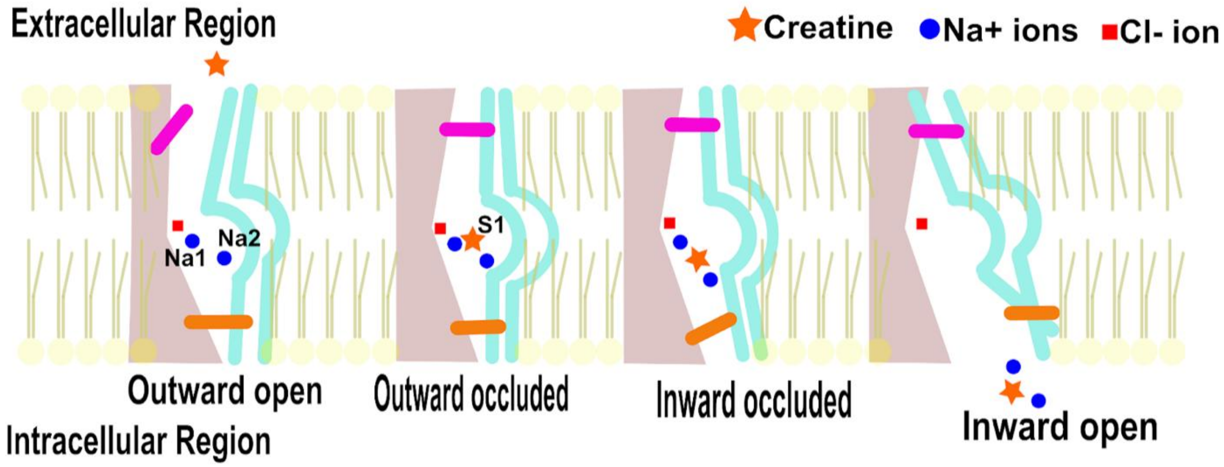

FIGURE S1 | Cartoon model illustrating creatine transport in CRT based on the alternating-access mechanism. S1 indicates the creatine-binding site. Na1 and Na2 are binding sites for Na1 and Na2 ions.

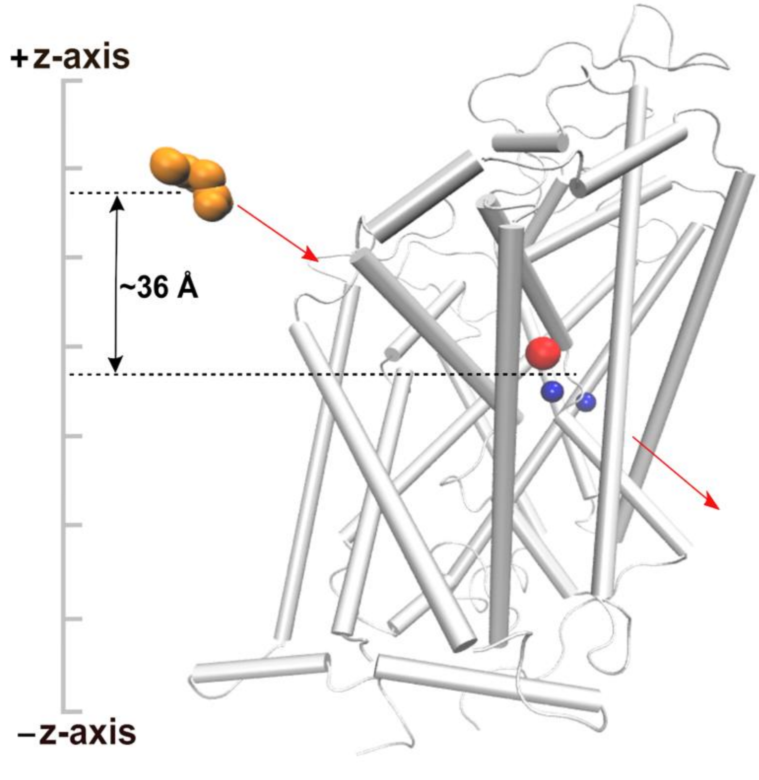

FIGURE S2 | Manual placement of creatine in the extracellular vestibule, ~36 Å from the S1 binding pocket in the outward open conformation of CRT. The protein is shown as a white cartoon, creatine as orange van der Waals spheres, and ions as spheres (red: Cl^-; blue: Na^+). The red arrow indicates the intended path for creatine.

All simulation trials.

To refine the protocol for simulating the full transport cycle, we conducted several exploratory simulations, starting with constant-force steered MD (cf-sMD), where the steering force was applied only to creatine (Trial 0), and then testing combinations of cf-sMD with tMD. These tests identified which methods could overcome the conformational barriers among the outward-open, outward-occluded, inward-occluded, and inward-open states, and also led to the correct sequential release of Na^+ ions and creatine (Na2 → creatine → Na1). Only the results from the final correct protocol are shown in the main text. The trials described below explain how consensus was reached on the final approach, including successful transitions and intermediate attempts that showed incomplete or non-physiological behaviors. While some analyses are also presented in the main article, they are provided here for continuity and improved readability. All simulations followed the parameters described in the 'Methods' in the main article unless otherwise specified. Trajectories were set to run for 500 ns; however, simulations that deviated from the expected release sequence were stopped at the point of deviation and not extended. Additionally, 500 ns was also chosen as a cutoff simulation time; if no additional expected event occurs until 500 ns, the simulation is terminated. This includes conformational changes from one state to another and the release time between species. Additionally, all distance analyses reported were based on center-of-mass (COM) measurements, with data sampled every 400 ps.

Trial 0: Steering creatine only in the outward-open conformation

As described in the main text, we first examined the effect of applying cf-sMD to creatine alone, using forces ranging from 5 to 100 pN. At 5 pN, creatine failed to enter the S1 site across all three replicas (Figure S3, A). We observed that the substrate fluctuated near the EC vestibule, engaging transiently with gate residues before becoming trapped in an alternative potential minimum. This force was not considered further. At 10 pN, creatine reliably reached the S1 site within 50 ns (40 ns, 45 ns, and 48 ns across the replicas) (Figure S3, B); however, EC gate closure remained incomplete (Figure S3, D, F). TM1b-TM9(up) distances remained unchanged or subsequently increased in two replicas, and only one trajectory showed a modest decrease after 400 ns (Figure S3, D). Similarly, TM6a-TM9(up) distances showed no consistent reduction across replicas (Figure S3, F). At 20 pN, creatine entered the S1 site within 50 ns (41 ns, 8 ns, and 24 ns across the replicas) (Figure S3, C), and one trajectory (replica 3, colored 'red') displayed a more pronounced and sustained decrease in both TM1b-TM9(up) (Figure S3, E) and TM6a-TM9(up) (Figure S3, G). Higher forces (>20 pN) were also tested; however, these rapidly transported creatine toward the intracellular side without the correct conformational changes and were therefore excluded. At this point, we also argued that replica 3 may represent the outward-occluded-like conformation for the CRT protein (discussed in the main article, section 2.1). Although replica 3 at 20 pN progressed toward outward occlusion from the outward-open conformation, it remained limited to this conformation without further transition. Once creatine reaches the S1 site, CRT is expected to transition from an outward-occluded to an inward-occluded

conformation; however, even after sufficient simulation time, we did not observe the expected transition. These observations led us to conduct the following subsequent trials.

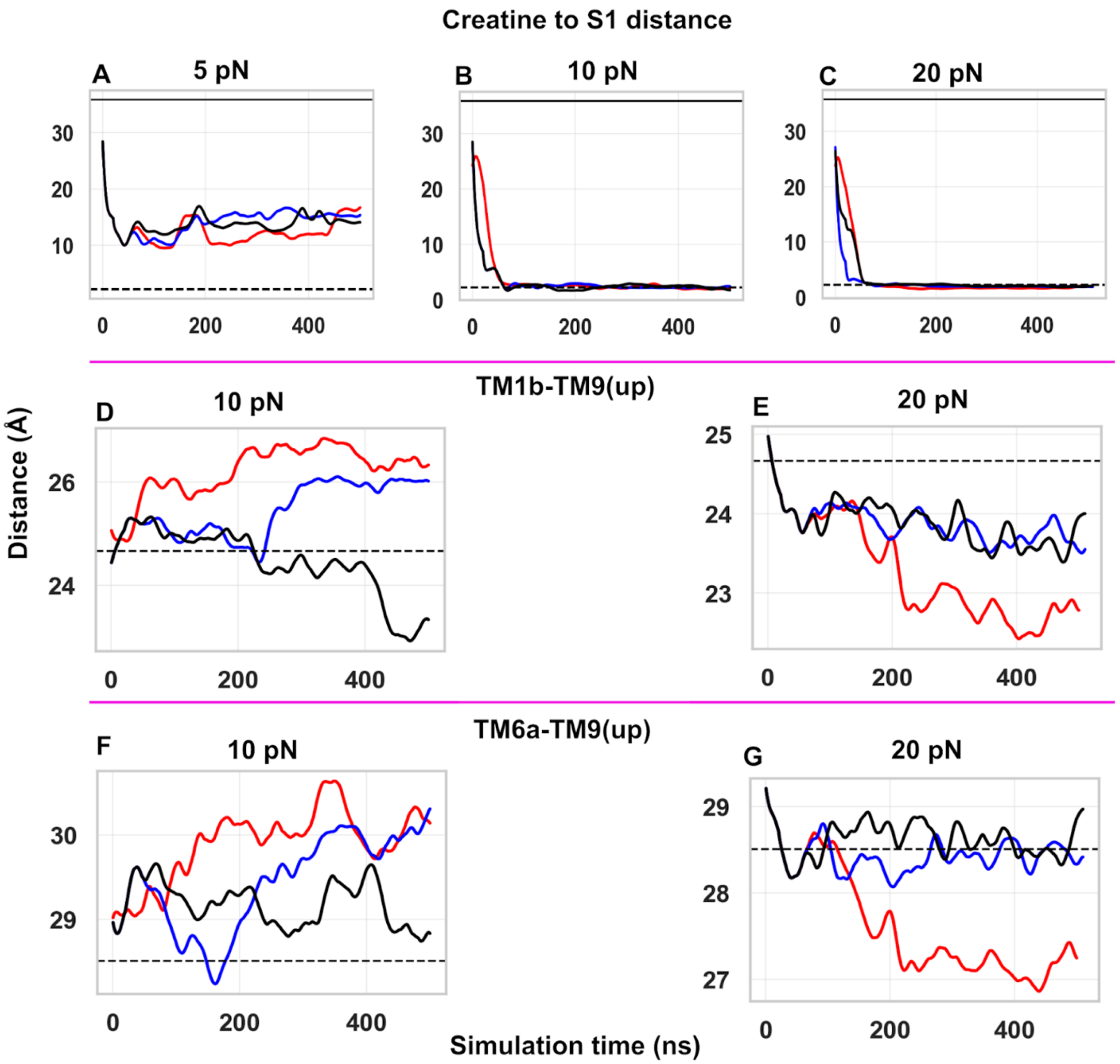

FIGURE S3 | Creatine entry into the S1 site and extracellular (TM1b/TM6a-TM9(up)) gating dynamics under different steering forces. This pertains to results obtained when the steering force was applied only to the creatine molecule. (A-C) COM distance between creatine and S1-site residues at 5 pN (**A**), 10 pN (**B**), and 20 pN (**C**), shown for three replicas (black, blue, red). The solid line indicates the corresponding distance in the outward-open homology model used as the starting structure; the dashed line indicates the distance in the inward-occluded crystal structure (PDB 9KRH)[1] shown for reference. At 5 pN, creatine does not enter the pocket; at 10 and 20 pN, it reaches S1 in all replicas and remains stably bound for the remainder of the simulation. (**D-E)** TM1b-TM9(up) COM distance under 10 pN (**D**) and 20 pN (**E**). The dashed line indicates the corresponding distance in the outward-open homology model. A reduction in this distance reflects extracellular-gate closure. At 20 pN, outward occlusion is observed in all replicas, with replica 3 (red) showing the most pronounced and sustained closure. **(F-G)** TM6a-TM9(up) COM distance under 10 pN (**F**) and 20 pN (**G**). The dashed line

indicates the corresponding distance in the outward-open homology model. TM6a shows limited early gating motion; modest reductions in replica 3 at 20 pN complement the TM1b-TM9(up) closure seen in panel E. Overall, TM6a exhibits less early movement relative to TM9(up) than TM1b does. The x-axis and y-axis are shared across all panels and represent simulation time (ns) and distance (Å), respectively. Additionally, the plotted traces correspond to locally averaged distances calculated over a 1-ns sliding window.

Trial 1: Simultaneous steering of creatine and Na^+ ions from the outward-occluded conformation

Following the simulation in trial 0, in which 20 pN transported creatine to S1, induced an outward-occluded conformation, and did not lead to inward occlusion, we examined whether co-steering both Na^+ ions (Na1 and Na2) with creatine would promote progression toward an inward-occluded conformation. Because creatine entered the S1 site within approximately 50 ns in the simulations steering creatine alone at 20 pN, we initiated simultaneous steering of creatine and both Na^+ ions at 150 ns across all three replicas and extended the simulations to 500 ns. Note that the Cl^- ion is hypothesized not to be transported1, so the steering force was not applied to this ion here and hereafter. Despite simultaneous steering of creatine and Na^+ ions, the observations remained essentially the same as with creatine alone. The system remained in a conformation similar to the outward-occluded conformation observed in trial 0, as reflected in the orientations of TM1a and Phe68 (compare Figure 1, F (main article) with Figure S4, Trial 1). A notable difference is seen in the orientation of the Phe68 ring; in this trial, the shift toward inward occlusion is slightly more pronounced than in trial 0. These results indicated that simultaneous steering of the substrate and ions also does not overcome the conformational barrier separating the outward-occluded and inward-occluded conformations. This reinforced the idea that simply steering creatine and ions is insufficient and that the barrier arises from protein conformational mechanics rather than ligand positioning or displacement. This motivated the introduction of tMD in subsequent trials to actively guide the protein through the remaining structural transitions. Taken together, these observations from trial 0 and trial 1 identified 20 pN as the minimal force that reliably transports creatine into the binding pocket and drives CRT toward an outward-occluded conformation, but this force alone is unable to overcome the conformational barrier separating the outward-occluded and inward-occluded conformations. Building on this rationale, the 150 ns structure from replica 3 at 20 pN in trial 0, which captured the most stable outward-occluded conformation, was selected as the starting point for the subsequent tMD-assisted trials discussed below.

Trial 2: tMD toward the inward-occluded conformation, followed by cf-sMD alone without tMD

Given that cf-sMD alone was insufficient to promote the transition from the outward-occluded to the inward-occluded conformation, we next introduced tMD to guide CRT from the outward-occluded to the inward-occluded conformation. Using the conformation at 150 ns from replica 3 at 20 pN, we initiated three independent simulations, each applying tMD toward the inward-occluded crystal structure (PDB 9KRH) for 50 ns while maintaining a cf-sMD force of 5, 10, or 20 pN on creatine and Na^+ ions (Na1 and Na2). As stated above, Na^+ ions are expected to be

released along with creatine; therefore, from this stage onward, steering was also applied to these ions (excluding Cl^{-} ions). Because tMD drives the protein across the conformational barrier separating the outward-occluded and inward-occluded conformations, we expected that a force lower than the initial 20 pN might be sufficient to drive substrate and ion movement toward the cytosolic side, so we included 5 and 10 pN in addition to 20 pN. After 50 ns, all tMD restraints were removed, and the simulations continued under cf-sMD alone, with the same applied force, for a total of 500 ns. By the end of the 50-ns tMD stage, the CRT conformation adopted an inward-occluded-like conformation consistent with the target structure, as reflected by the bend in TM1a (Figure S4, Trial 2, green and magenta ribbons almost overlap). However, following removal of tMD, TM1a, which had bent appropriately during tMD, relaxed back toward its pre-tMD orientation (Figure S4, Trial 2, blue ribbon and red ribbon almost overlap), likely re-establishing the intracellular gate interactions that characterize the outward-occluded conformation. As a result, the pathway is blocked, preventing the release of creatine or Na^{+} even after 500 ns. We observed similar results for all forces across all replicas. Although the tMD guided the system to the inward-occluded conformation, it was not sufficiently stabilized to persist after the restraint was removed; thus, upon lifting the bias, the helix returned to its pre-tMD position. While not shown here, we also did not observe any notable difference in the orientation of Phe68. This indicates that the inward-occluded state sampled under tMD had not yet become an energetically stable conformation under standard MD and the applied constant force alone. Because of this relaxation, cf-sMD by itself was unable to drive the subsequent inward-occluded to inward-open conformation or produce the expected release of creatine and the Na^{+} ions. These observations motivated the introduction of a second tMD stage discussed below.

Trial 3: tMD toward the inward-open conformation followed by cf-sMD alone

In this trial, we applied a two-stage tMD protocol. Using the conformation at 150 ns from replica 3 at 20 pN, we initiated three independent simulations in which cf-sMD forces of 5, 10, or 20 pN were applied simultaneously with tMD. The first stage of tMD included 50 ns toward the inward-occluded crystal structure (PDB 9KRH), followed by 50 ns toward the inward-open crystal structure (PDB 9KR7)[1], constituting the second stage of tMD. This combined procedure successfully produced an inward-open-like conformation, characterized by the expected forward bending of TM1a (Figure S4, Trial 3, left, green ribbon) and by rotation of Phe68 toward its inward-open orientation (Figure S4, Trial 3, right, green licorice). As in Trial 2, once the two-stage tMD simulations were completed, all tMD restraints were removed, and the simulations continued under cf-sMD alone for up to 500 ns for the remainder of the trajectory. The overall behavior was similar to that of Trial 2: TM1a partially relaxed toward its pre-tMD orientation, though the degree of relaxation was less pronounced. However, a notable change was observed in Phe68. While its phenyl ring adopted the inward open conformation during tMD (green licorice), it tended to rotate back toward the orientation present before tMD (green shifted back to blue licorice), and its plane of orientation was similar to that in the starting outward occluded structure (red). Although the planes don't completely overlap, the tendency of the shift is clearly observed. Because the rotation

of Phe68 is critical for creating an open intracellular pathway and enabling the release of ions and substrate1, its reversal after tMD removal effectively prevented progression of the transport cycle. These results demonstrate that even when the system is driven into an inward-open-like conformation, releasing tMD allows TM1a and Phe68 to revert to their earlier orientations, thereby re-closing the intracellular gate and preventing release. Note that we observed similar results for all the forces across all the replicas, except for one replica at 20 pN. For this replica, we observed that Na2 and creatine were released during the second stage of tMD. As this outcome was observed in only one of three replicas and was not statistically significant, the trajectory was excluded from further consideration in this trial. This observation motivated Trial 4, in which the inward open conformation was preserved by maintaining tMD throughout the simulation.

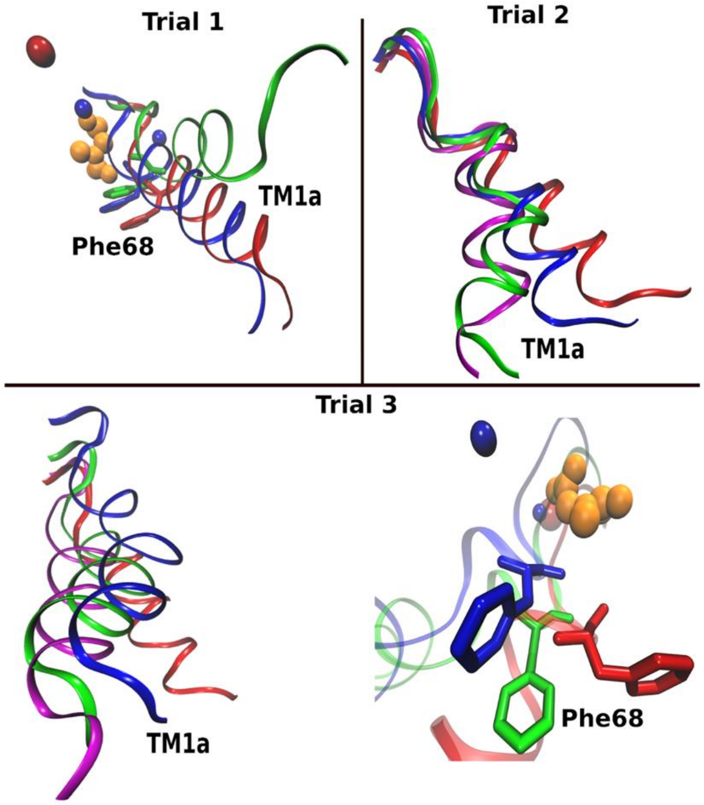

FIGURE S4 | TM1a conformational changes across representative hybrid cf-sMD and tMD simulations. Creatine is shown as orange van der Waals spheres, and ions as spheres (red, Cl^-; blue, Na^+). Results are shown for a representative replica. (**Trial 1)** TM1a conformational changes during cf-sMD with steering forces applied simultaneously to creatine and sodium ions. Structures correspond to the end of cf-sMD (blue), the fully equilibrated outward-open homology model used as the starting structure (red), and the inward-occluded crystal structure (green; PDB 9KRH). The observed behavior is comparable to that obtained when steering creatine alone (Figure 2, F, main text). (**Trial 2)** TM1a conformations from different stages of the simulation: the outward-occluded starting conformation at 150 ns before tMD (red), the structure at the end of 50 ns tMD toward the inward-occluded state (green), the conformation after removal of tMD restraints during subsequent cf-sMD (blue), and the inward-occluded crystal structure (magenta; PDB 9KRH). TM1a bends toward the inward-occluded orientation during tMD but relaxes toward the pre-tMD conformation after restraint release. (**Trial 3,**

Left) TM1a conformations from the outward-occluded state at 150 ns before tMD (red), the end of second-stage tMD toward the inward-open state (green), the post-tMD cf-sMD conformation (blue), and the inward-open crystal structure (magenta; PDB 9KR7). TM1a bends toward an inward-open orientation during tMD but partially relaxes afterward. (**Trial 3, Right)** Superposition of Phe68 with the same color scheme, revealing a noticeable shift in side-chain orientation.

Trial 4: Continuous tMD with simultaneous cf-sMD (final protocol)

The final trial tested whether retaining tMD throughout the inward-open conformation would prevent the partial collapse of the intracellular gate observed in earlier trials and lead to releases. Starting from the 150 ns structure of the outward occluded replica 3 obtained at 20 pN, we initiated simulations in which cf-sMD forces of 5, 10, or 20 pN were applied simultaneously with tMD. As in Trial 3, each simulation included 50 ns of tMD toward the inward-occluded crystal structure, followed by 50 ns of tMD toward the inward-open crystal structure. However, unlike in the previous trials, we did not lift tMD after the second-stage tMD. Instead, the tMD restraints were maintained for the remainder of each trajectory, and the same cf-sMD forces (5, 10, or 20 pN) continued to be applied simultaneously with tMD for the remaining simulation. Unlike the earlier trials, this protocol led to the release of Na2, creatine, and Na1, allowing us to evaluate both the order and timing of release. Also, in Trial 4, we increased the number of replicas from three (used in the exploratory trials above) to five per force condition to make the observed trends more robust for release events and their release times.

Under these conditions, the intracellular gate remained open, and TM1a did not relax toward its pre-tMD orientation, unlike in Trials 2 and 3, thereby leading to the release of the species. The release times for Na2, creatine, and Na1 in each replica and force condition are summarized in Table T1. All release times reported in Table T1 are measured from the start of the tMD protocol (t = 0 at tMD initiation), i.e., after the 150 ns outward occluded starting structure was selected. At 10 and 20 pN, creatine was transported toward the cytosolic side prematurely in multiple trajectories, releasing before Na2; these runs violated the expected release sequence and were terminated at the point of departure (as detailed in Table T1). In addition, several trajectories at 10 and 20 pN produced no release events within the ~700 ns window (nothing out, Table T1). In contrast, the lowest force (5 pN) produced the expected sequential release order (Na2 → creatine → Na1) in four of five replicas, with the remaining replica showing Na2 release without subsequent release of creatine or Na1 within the simulated time. Together, these results identified 5 pN as the optimal steering force for this final protocol, as it reproducibly ensures the expected sequential release of substrate and ions while still enabling release within accessible simulation times (the maximum release time is ~700 ns). Therefore, 5 pN was selected for the subsequent production simulations and analyses of the intracellular release pathway.

Note that, for trajectories in which no release event occurred (nothing out), simulations were terminated at ~700 ns, based on the observation that complete release (Na2, creatine, and Na1) occurred within ~700 ns in the successful 5 pN trajectories. Also, a criterion of 500 ns was set for interevent gaps; i.e., if the release of one species from the already-released species does not occur

within 500 ns, the simulation is discarded as a kinetic trap. It should also be noted that the release sequence has been proposed only for CRT, based on analogy with other SLC6 transporters and supported by available inward-occluded and inward-open crystal structures; however, a continuous simulation of the complete transport process that captures this sequence has not previously been reported. Notably, the same steering force was applied to both ions and creatine simultaneously, without introducing a differential bias between species, yet the expected release order emerged reproducibly at low force. These observations further support both the hypothesized transport mechanism and the reliability of our simulation protocol under the chosen force condition. Additionally, the reason the lower force works during release is discussed in the main text (Discussion).

These results indicate that maintaining tMD throughout the inward open conformation is essential for the intracellular opening required for substrate and ion release. In our simulations, TM1a bending and intracellular gate opening did not persist upon removal of tMD, suggesting that the system had not yet crossed the full conformational barrier separating the inward-occluded and inward-open conformations. By keeping tMD active, this barrier was effectively overcome, allowing the transporter to adopt and maintain an inward-open-like geometry long enough for release events to occur, while the applied cf-sMD force remained minimal. Collectively, these trials established that both continuous tMD and a minimal pulling force are required to ensure the release events. However, it should be acknowledged that tMD is used to overcome a known kinetic barrier and that the release mechanism is studied within the targeted conformation.

To further quantify the intracellular release process under the optimal 5 pN condition, we examined the trajectory-resolved release times of Na2, creatine, and Na1 across the five simulations (Figure 3 (main article); Table T1). While the main text summarizes the overall release behavior, the analysis below provides a detailed breakdown of the timing of individual release events and the intervals separating them. Also, some results have been repeated to ensure better readability and clarity. Here, we report the individual release times of Na2, creatine, and Na1 (with Na1 release also indicating the overall completion time for the release stage), as well as the time gaps between the release of each species (Na2 to creatine, creatine to Na1, and Na2 to Na1).

Across the five trajectories simulated at 5 pN, intracellular release consistently followed the expected sequential order, although the precise timing of individual events varied between trajectories. In replicas 1-4, all three release events occurred within the simulation window, and the ordering of events remained invariant. Absolute release times spanned a broad range: Na2 released between 145-558 ns, creatine between 192-657 ns, and Na1 between 401-682 ns. The medians across Replicas 1-4 were 246 ns (Na2), 422 ns (creatine), and 675 ns (Na1), illustrating a clear stepwise progression in a typical trajectory, even though individual replicas can proceed substantially faster or slower. A closer inspection of individual trajectories shows that the timing differences follow recognizable patterns rather than occurring randomly. Replica 4 is consistently fast across all three events (145/192/401 ns in the order Na2/creatine/Na1), whereas replica 2 is systematically slower for Na2 and creatine (558/657 ns) and remains late for Na1 (672 ns).

Replicas 1 and 3 show intermediate behavior. Notably, although Na2 releases much earlier in replica 3 than in replica 1 (171 vs 320 ns), creatine and Na1 release at similar times in the two trajectories (replica 1: 320/415/682; replica 3: 171/429/678). This comparison indicates that an earlier Na2 release does not necessarily propagate to earlier creatine or Na1 release in the same trajectory.

To better understand the progression between sequential steps, we also examined the inter-event intervals between release events (Table T1, Supporting Information 1). In replicas 1-4, the Na2-to-creatine gap is typically short (47–99 ns in replicas 1, 2, and 4), indicating that once Na2 leaves the transporter, creatine release often follows within a relatively short time window. However, replica 3 shows a substantially longer Na2-to-creatine interval (258 ns), indicating that the intermediate state following Na2 exit can persist for hundreds of nanoseconds before creatine becomes release-competent. The creatine-to-Na1 interval is generally longer (~200-270 ns in replicas 1, 3, and 4), consistent with Na1 frequently representing the final step of the intracellular release process while the pathway continues to reorganize. Replica 2 represents a notable exception: creatine and Na1 release occur within 15 ns of one another (657 vs 672 ns), implying that in this trajectory, the final step is not strongly rate-limiting once creatine exits, even though the overall process remains slow in absolute time.

Taken together, these comparisons indicate that the principal source of variability between trajectories is not a change in event ordering but rather trajectory-dependent differences in the lifetimes of intermediate states that separate successive release steps. The fifth trajectory provides an additional constraint on this interpretation. In Replica 5, only Na2 was released (180 ns), while creatine and Na1 remained bound within the simulated time window. This outcome is consistent with the ordering observed in the complete trajectories (Na2 leaves first), but it also highlights that Na2 release alone is not sufficient to guarantee completion of the downstream steps within the accessible timescale. The system can therefore reach a state in which Na2 escapes, while creatine and Na1 remain trapped in long-lived bound conformations, indicating the presence of additional kinetic barriers that may or may not be crossed along a given trajectory, even under identical forcing conditions.

Finally, this trajectory-resolved analysis further supports selecting 5 pN for the production simulations. Across the force conditions tested, 5 pN most consistently produced discrete, interpretable release events while preserving the expected sequential pathway. In contrast, 10 and 20 pN more frequently produced either non-sequential behavior (e.g., early creatine release before Na2) or incomplete release within the simulation window, indicating that higher forces can disrupt the expected pathway rather than simply accelerating it. The 5 pN steering force, therefore, provides the most reliable basis for subsequent mechanistic analysis of the intracellular release process.

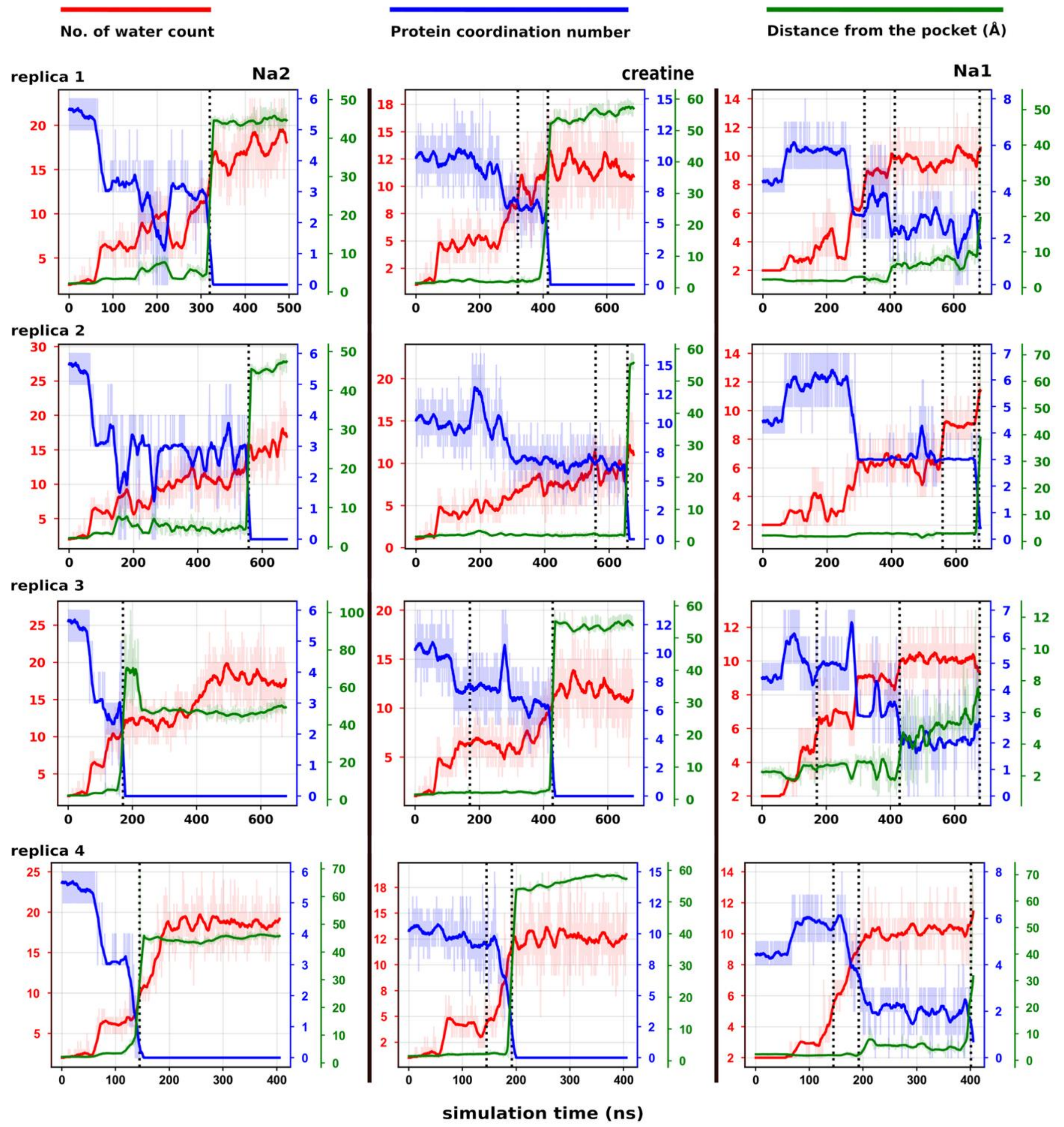

FIGURE S5 | Local hydration and protein coordination changes analysis for intracellular release events at 5 pN. Time series are shown for Na2 (left column), creatine (middle column), and Na1 (right column) across Replicas 1-4 (rows). For each species, the number of water molecules within 3.5 Å (red), the number of nearby protein polar donor atoms (O, N, S) within 3.5 Å (blue), and the COM distance between the species and residues within 3.5 Å of the species (green) are plotted as a function of simulation time. Dotted vertical lines mark the corresponding release times reported in Figure 2 in the main article and Table T1.

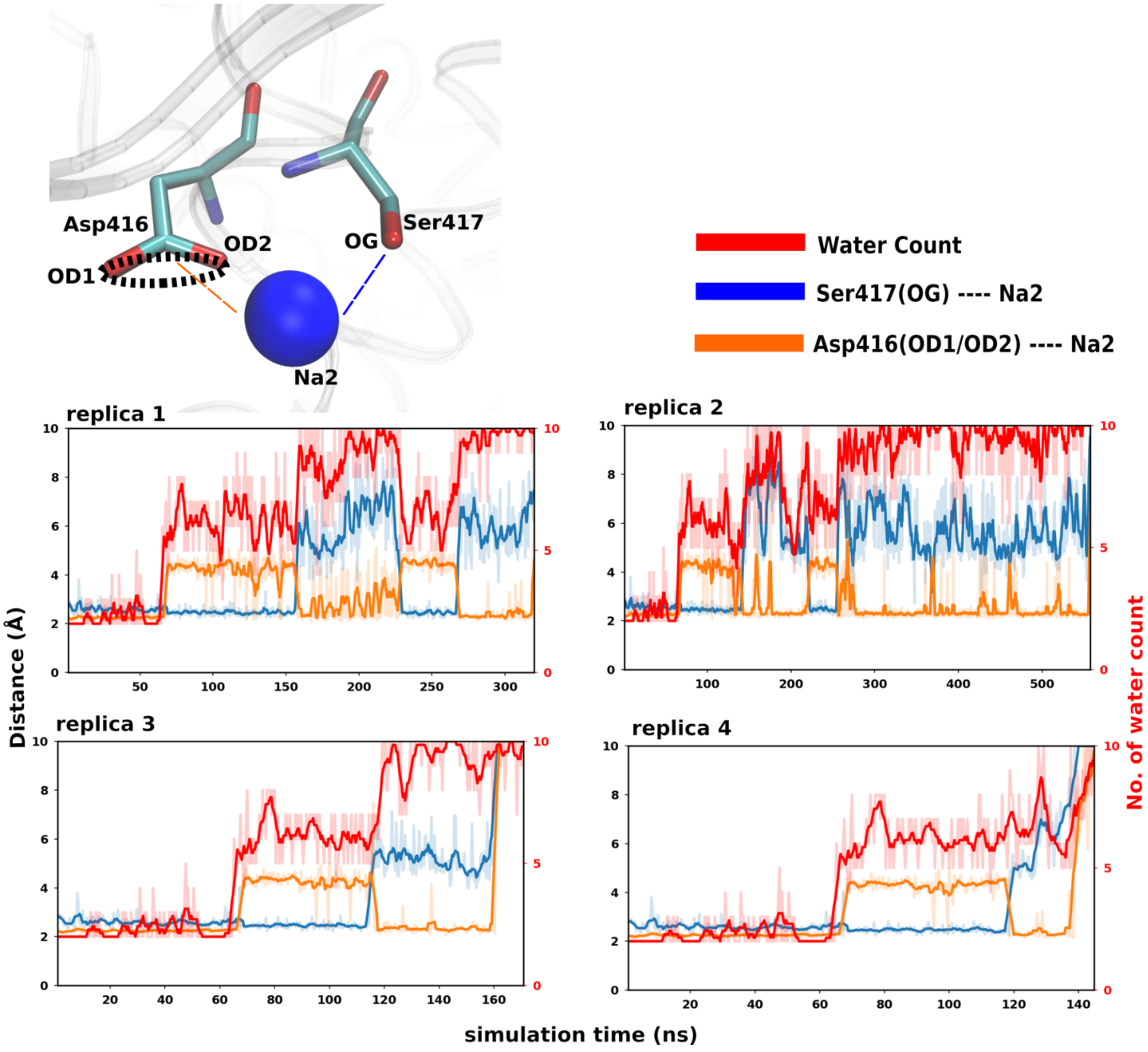

FIGURE S6 | Hydration and anchor-distance analysis for Na2 across all four replicas. The blue sphere represents Na2. Distances are shown for Ser417(OG)-Na2 (blue) and Asp416(OD1/OD2)-Na2 (orange), along with the number of water molecules within 3.5 Å of Na2 (red). For Asp416, the plotted value is the minimum of the OD1-Na2 and OD2-Na2 distances at each frame. All panels share a common x-axis of simulation time (ns). Distances are reported on the left y-axis (Å, black), and water counts on the right y-axis (red). The complete list of protein-Na1 anchor pairs is provided in Table 2.

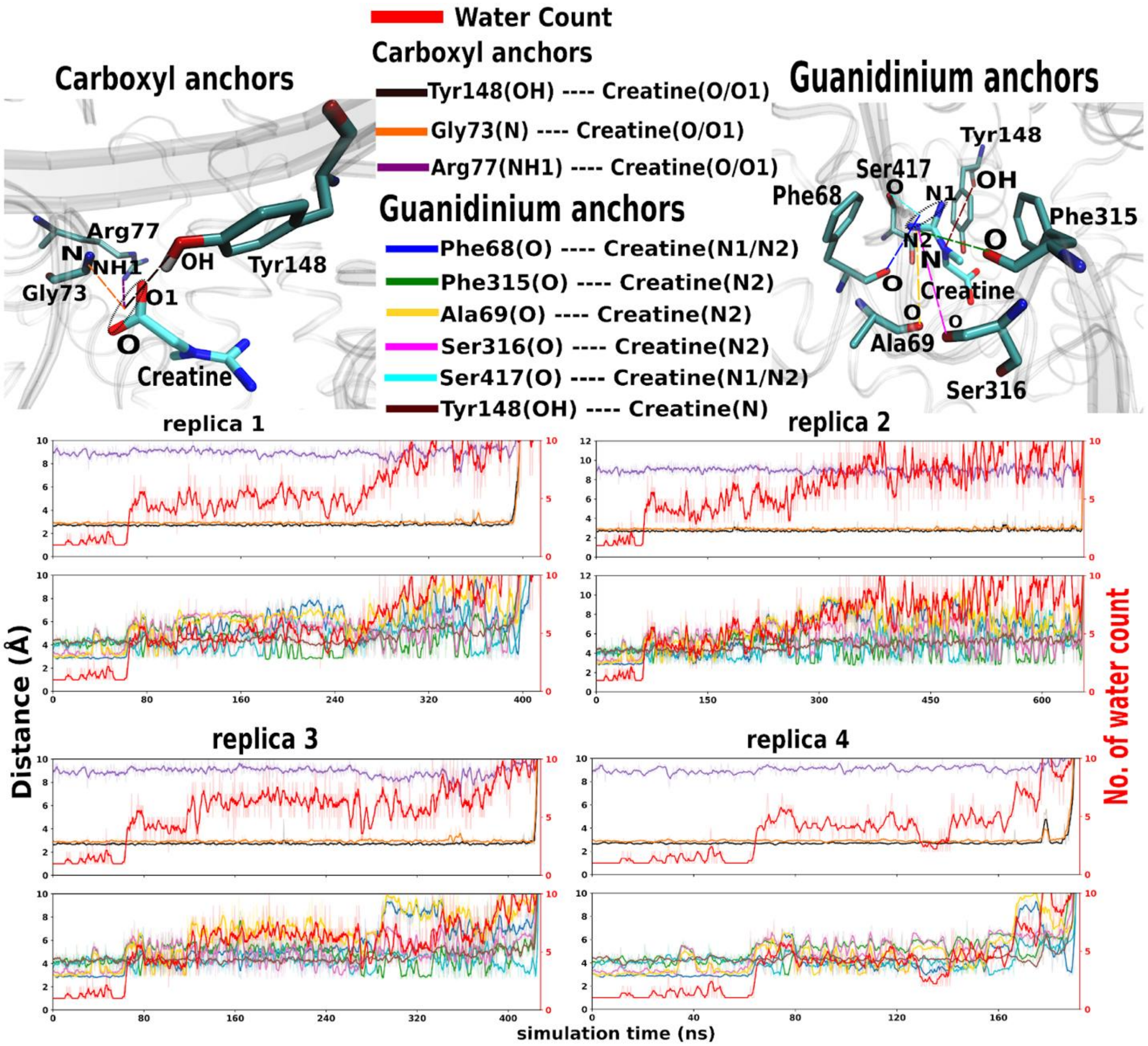

FIGURE S7 | Hydration and anchor-distance analysis for creatine across all four replicas. Anchor-distance time series are separated into carboxylate-side (upper) and guanidinium-side (lower) contacts, each plotted with the number of water molecules within 3.5 Å of creatine (red). Carboxylate anchors (upper): Tyr148(OH)-creatine(O/O1) (black), Gly73(N)-creatine(O/O1) (orange), and Arg77(NH1)-creatine(O/O1) (purple). Guanidinium anchors (lower): Phe68(O)-creatine(N1/N2) (blue), Phe315(O)-creatine(N2) (green), Ala69(O)-creatine(N2) (gold), Ser316(O)-creatine(N2) (magenta), Ser417(O)-creatine(N1/N2) (cyan), and Tyr148(OH)-creatine(N) (brown). For anchors involving equivalent atoms (e.g., O/O1 or N1/N2), the plotted distance is the minimum at each frame. All panels share a common x-axis of simulation time (ns). Distances are reported on the left y-axis (Å, black), and water counts on the right y-axis (red). The complete list of protein-creatine anchor pairs is also provided in Table 2.

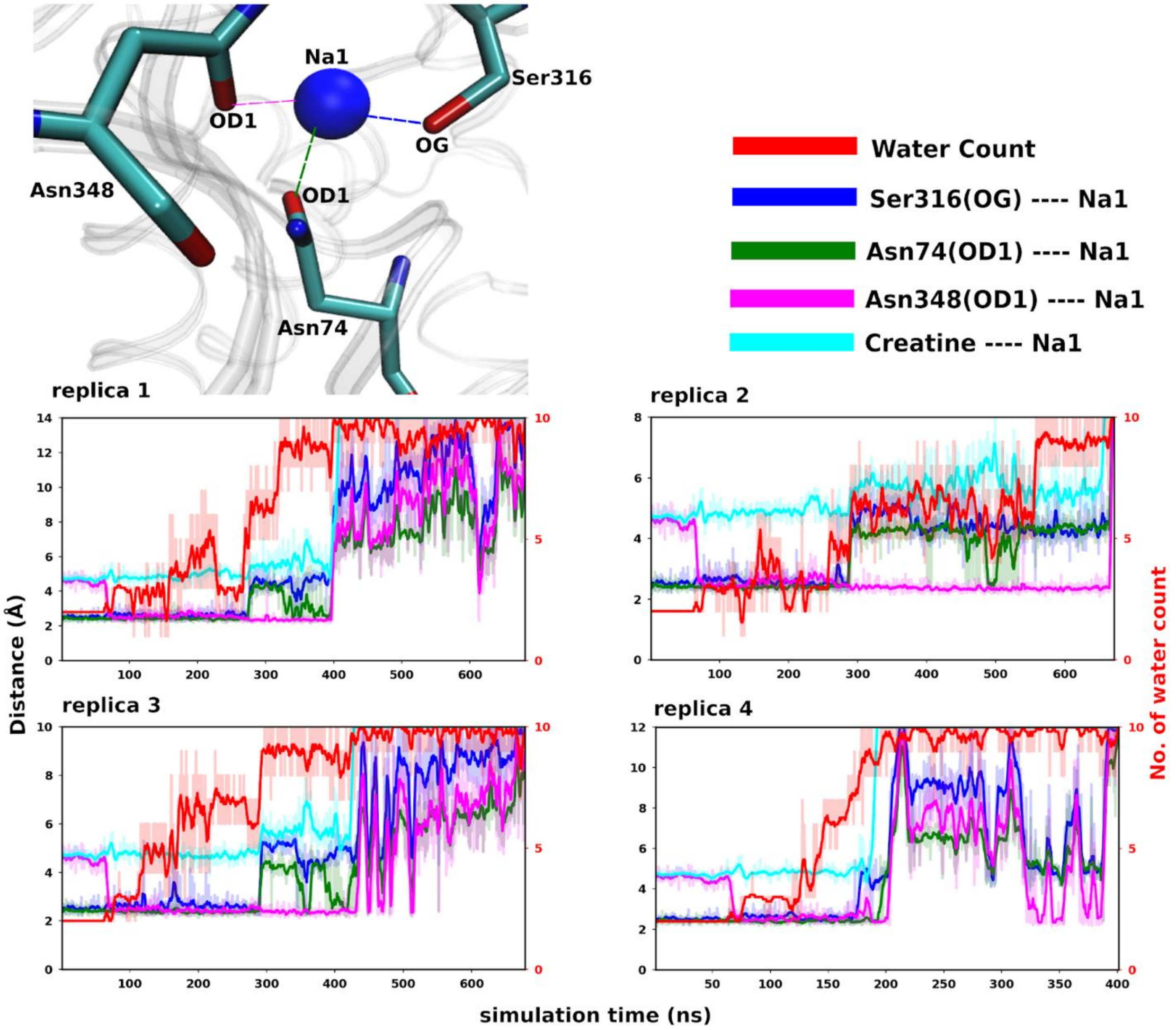

FIGURE S8 | Hydration and anchor-distance analysis for Na1, shown across all four replicas. The blue sphere represents Na1. Distances are shown for Ser316(OG)-Na1 (blue), Asn74(OD1)-Na1 (green), Asn348(OD1)-Na1 (magenta), and creatine-Na1 (cyan), along with the number of water molecules within 3.5 Å of Na1 (red). All panels share a common x-axis of simulation time (ns). Distances are reported on the left y-axis (Å, black), and water counts on the right y-axis (red). The complete list of protein-Na1 anchor pairs is provided in Table 2.

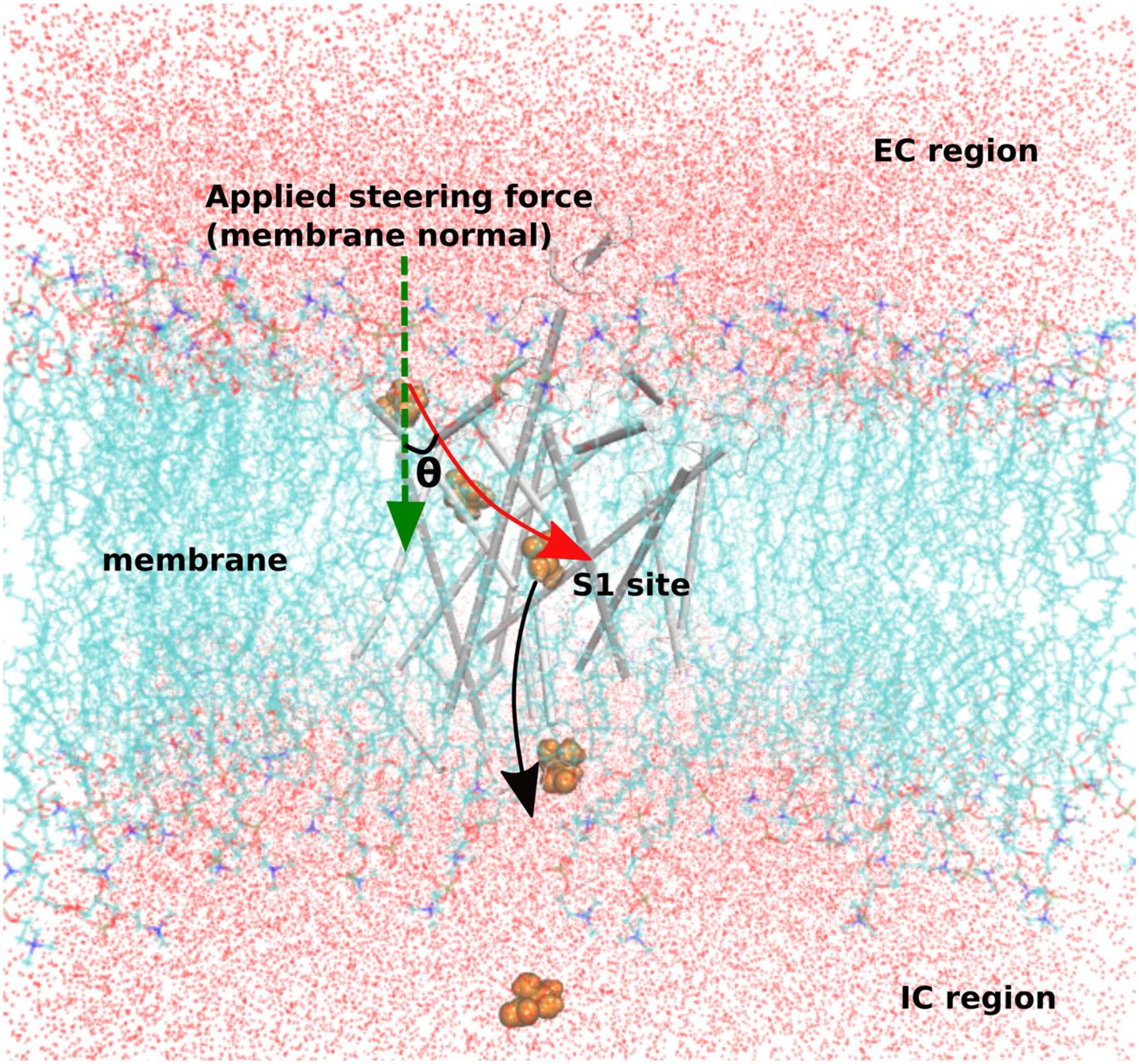

FIGURE S9 | Force projection along the creatine extracellular entry and intracellular release. A constant steering force is applied to the substrate along the membrane normal (green dashed arrow). During extracellular (EC) entry, creatine follows a curved vestibular pathway toward the S1 binding site (red arrow). Because the applied force is not colinear with the reaction coordinate, only the projected component of the force ($F_{\text{effective}} = F\cos\theta$) contributes to motion along the pathway. In contrast, during intracellular (IC) release, substrate movement occurs approximately along the direction of the applied force (black arrow), reducing the required force magnitude.

TABLE T1 | Release times (ns) for Na2, creatine, and Na1 across five replicas under constant steering forces of 5, 10, and 20 pN. Each cell reports release times in the order Na2 / creatine / Na1. 'Not out' indicates that the corresponding species did not release within the simulated time. Simulations were terminated upon violation of the expected sequential release order, and trajectories with no release events were terminated at ~700 ns. All release times reported in Table T1 are measured from the start of the tMD protocol (t = 0 at tMD initiation), i.e., after the 150 ns outward occluded starting structure was selected.

**Force (pN)**	**Replica 1 (ns)**	**Replica 2 (ns)**	**Replica 3 (ns)**	**Replica 4 (ns)**	**Replica 5 (ns)**
**5**	320 / 415 / 682	558 / 657 / 672	171 / 429 / 678	145 / 192 / 401	180 / Not out / Not out (only Na2 out)
**10**	Not out / Not out / Not out (nothing out)	Not out / 436 / Not out (Creatine out first so stopped)	355 / 371 / 379	Not out / 442 / Not out (Creatine out first so stopped)	217 / Not out / Not out (only Na2 out)
**20**	Not out / Not out / Not out (nothing out)	Not out / Not out / Not out (nothing out)	376 / 378 / 530	Not out / 156 / Not out (Creatine out first so stopped)	Not out / Not out / Not out (nothing out)

**Median Release time for 5 pN**	Na2 = 245 ns	Creatine = 422 ns	Na1 = 675 ns

**Replica (5pN)**	Δt (Na2→Cr) ns	Δt (Cr→Na1) (ns)	Δt (Na2→Na1) (ns)
**1**	95	267	362
**2**	99	15	114
**3**	258	249	507

**4**	47	209	256
**Median (Δt)**	97	229	309

TABLE 2 | Protein-substrate anchor contacts used for distance and stage analyses. Protein-substrate anchor contact pairs. Creatine anchors are grouped according to interactions with the carboxylate moiety (O/O1) or the guanidinium moiety (N, N1, N2). For anchors involving equivalent atoms, the reported distance corresponds to the minimum of the listed atom pairs (for example, O/O1, OD1/OD2, or N1/N2). Anchor sets consisted of 2 contacts for Na2, 8-10 contacts for creatine, and 3 contacts for Na1.

Species	Anchor Groups	Anchor Residue Pairs
**Na2**	-	Ser417(OG)-Na2, Asp416(OD1/OD2)-Na2
**Creatine**	**Carboxyl anchors**	Tyr148(OH)-Creatine(O/O1), Gly73(N)-Creatine(O/O1), Arg77(NH1)-Creatine(O/O1)
**Creatine**	**Guanidinium anchors**	Tyr148(OH)-Creatine(N), Phe68(O)-Creatine(N1/N2), Ala69(O)-Creatine(N2), Phe315(O)-Creatine(N2), Ser316(O)-Creatine(N2), Ser417(O)-Creatine(N1/N2)
**Na1**	-	Ser316(OG)-Na1, Asn74(OD1)-Na1, Asn348(OD1)-Na1

TABLE 3 | Ensemble definitions based on hydration and anchor engagement. Criteria used to classify simulation frames into different ensemble stages for Na2, creatine, and Na1 during the release simulations. N_w is the number of water molecules within 3.5 Å of the species. N_a is the number of engaged protein-substrate/ions anchor contacts if the distance between them is <= 3.5 Å.

**Ensemble**	**Na2**	**Creatine**	**Na1**
**E1 (Tightly Bound)**	N_w <= 4 & N_a = 2	N_w <= 4 & N_a >= 3	N_w <= 4 & N_a >= 2
**E2 (Bound Intermediate)**	N_w <= 6 & N_a = 1	N_w <= 6 & N_a = 2	N_w <= 6 & N_a = 1-2
**E3 (Hydrating)**	N_w = 5-8 & N_a <= 1	N_w = 5-8 & N_a <= 1	N_w = 5-8 & N_a <= 1
**E4 (Released)**	N_w >= 9 & Na = 0	N_w >= 9 & Na <= 0-1	N_w >= 9 & Na <= 0-1

REFERENCES

1 Chen, J., Zhang, Y., Chen, N., Ge, J. & Yu, J. Transport and inhibition mechanisms of human creatine transporter. *Cell Discovery* **11**, 43 (2025).
